# Supplementary material for: VHL synthetic lethality screens uncover CBF-β as a negative regulator of STING
Source: Nat Commun. 2026 Mar 12;17:3841. doi: 10.1038/s41467-026-70517-w (PMC13121600; doi:10.1038/s41467-026-70517-w)
Supplement: Supplementary file 1 — Supplementary Information [file 41467_2026_70517_MOESM1_ESM.pdf]

Supplementary Information:

VHL synthetic lethality screens uncover CBF-β as a negative regulator of STING

Supplementary Figures 1-9

Supplementary Tables 1-2

Supplementary References

Supplementary Fig. 1

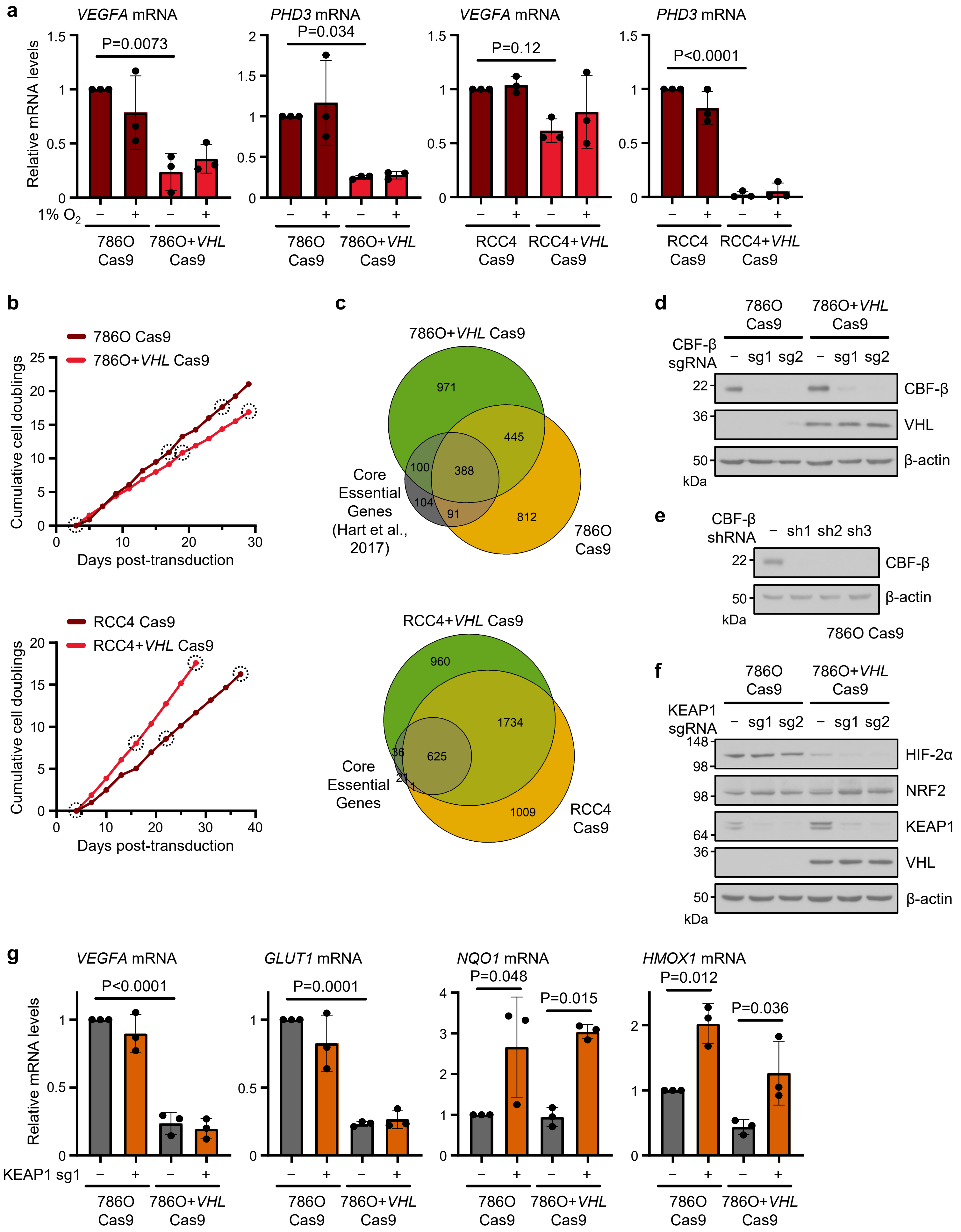

Supplementary Fig. 1. CRISPR/Cas9 screening in ccRCC cell lines reveals *VHL*-associated synthetic lethality of the transcription factors CBF-β and NRF2

(**a**) Expression of HIF target genes in ccRCC cells and paired VHL-reconstituted cells after culture at 1% or 21% O_2 for 24 hours. n=3 biologically independent replicates. Mean ± SD. Two-way ANOVA.

(**b**) Cumulative cell doublings from day 3 (786O screen) or day 4 (RCC4 screen) in CRISPR/Cas9 screens. Dotted circles indicate the analysed samples.

(**c**) Efficient identification of essential genes in CRISPR/Cas9 screens. Venn diagrams of essential genes which dropout between early and late timepoints identified by BAGEL2 with FDR<0.05, compared to a reference set of Core Essential Genes[1].

(**d-f**) Immunoblots of 786O Cas9 and 786O+*VHL* Cas9 cells transduced with sgRNAs targeting CBF-β (**d**), shRNAs targeting CBF-β (**e**), and sgRNAs targeting KEAP1 (**f**). sgRNA vectors were doxycycline-inducible, and cells were treated with 100 ng/ml doxycycline prior to analysis. Controls were transduced with an empty sgRNA expression vector (**d,f**), or a scrambled shRNA sequence (**e**). Immunoblots representative of 3 independent experiments.

(**g**) *KEAP1* knockout induces NRF2, but not HIF, activation. qPCR analysis of HIF targets (*VEGFA* and *GLUT1*) and NRF2 targets (*NQO1* and *HMOX1*) in cells transduced with an sgRNA targeting KEAP1. n=3 biologically independent replicates. Mean ± SD. Two-way ANOVA.

Source data are provided as a Source Data file.

Supplementary Fig. 2

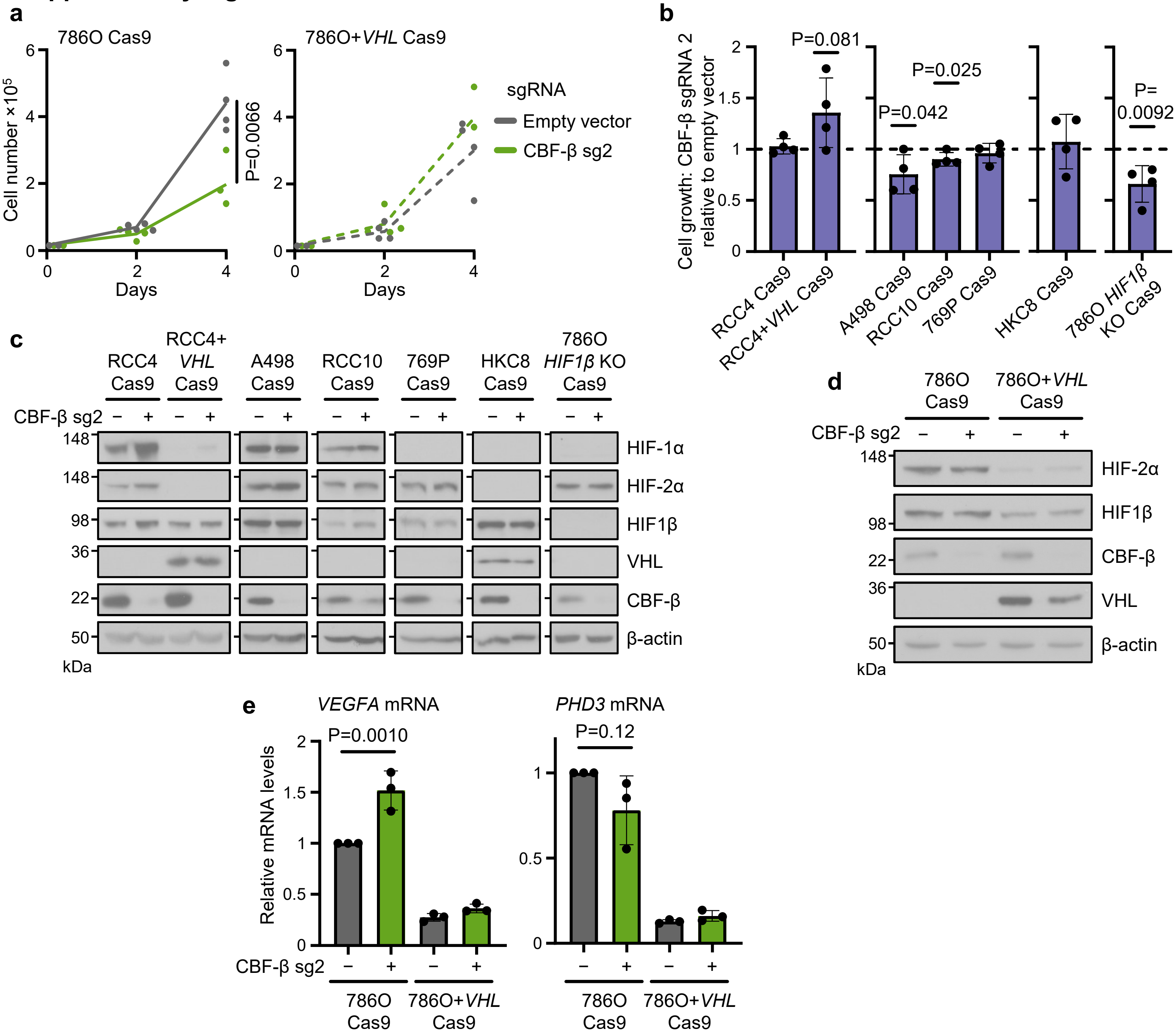

Supplementary Fig. 2. *CBFB* is synthetic lethal with *VHL* but does not affect HIF signalling

(**a**) Proliferation assay of 786O Cas9 and 786O+*VHL* Cas9 cells transduced with an sgRNA targeting CBF-β (CBF-β sg2), or an empty vector control. n=4 biologically independent replicates. Two-way ANOVA of cell number at day 4.

(**b**) Proliferation assay of ccRCC cells (RCC4, A498, RCC10 and 769P), renal proximal tubule epithelial cells (HKC8), and clonal *HIF1β*-deficient 786O cells and *VHL*-reconstituted RCC4 cells, transduced with CBF-β sg2. Cell numbers at day 4 plotted relative to control cultures transduced with an empty vector. n=4 biologically independent replicates. Mean ± SD. Unpaired *t*-test.

(**c**) Representative immunoblot of transductions assayed in **Supplementary Fig. 2b**. n=3 biologically independent replicates.

(**d,e**) *CBFB* does not regulate HIF activity in 786O Cas9 cells. 786O Cas9 and 786O+*VHL* Cas9 populations were transduced with CBF-β sg2 or an empty vector control and assayed by immunoblot (**d**), and by qPCR of HIF target genes (**e**). n=3 biologically independent replicates for **d** and **e**. Mean ± SD. Two-way ANOVA.

Source data are provided as a Source Data file.

Supplementary Fig. 3

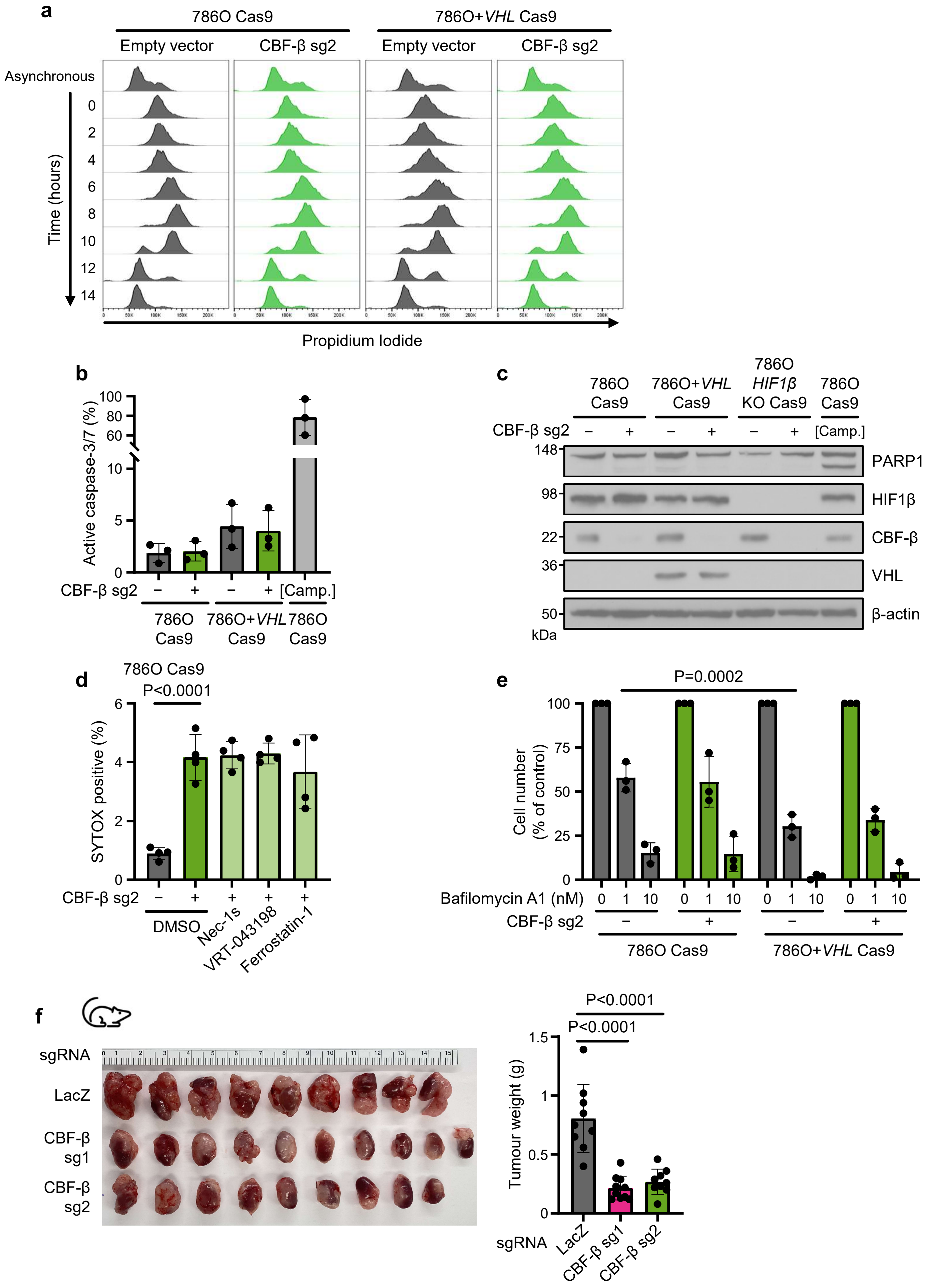

Supplementary Fig. 3. *CBFB* knockout induces cell death in *VHL*-deficient cells

(**a**) Rate of cell cycle progression in 786O Cas9 and 786O+*VHL* Cas9 cells transduced with CBF-β sg2 or an empty vector control. Cells were synchronised with a double thymidine block, and fixed for propidium iodide staining and flow cytometry every two hours following release. An asynchronous control is provided for comparison. Representative of 3 biologically independent replicates.

(**b**) Proportion of 786O Cas9 and 786O+*VHL* Cas9 cells exhibiting caspase-3/7 activity following transduction with CBF-β sg2 or an empty vector control, as detected by the CellEvent Caspase-3/7 Green flow cytometry reagent. [Camp.]: cells treated with 24 hours 100 μM Camptothecin as a positive control for apoptosis. n=3 biologically independent replicates. Mean ± SD. Two-way ANOVA.

(**c**) PARP1 cleavage in cells transduced with CBF-β sg2 or an empty vector control. Lower band on PARP1 membrane represents the cleaved form of PARP1. [Camp.]: 786O Cas9 cells treated with 10 μM Camptothecin as a positive control for apoptosis. Immunoblot representative of 3 biologically independent replicates.

(**d**) 786O Cas9 cells transduced with CBF-β sg2 or an empty vector control, and treated for 48 hours with 50 μM Necrostatin-1s (Nec-1s), 25 μM VRT-043198, 2 μM Ferrostatin-1 or the DMSO vehicle. Cells were assayed by flow cytometry using SYTOX AADvanced dead cell stain. n=4 biologically independent replicates. Mean ± SD. Two-way ANOVA.

(**e**) Cell number of 786O Cas9 and 786O+*VHL* Cas9 cells treated with Bafilomycin A1 at the indicated dose for 48 hours, normalised to cells treated with the DMSO vehicle. n=3 biologically independent replicates. Mean ± SD. Two-way ANOVA.

(**f**) Left kidneys harvested from mice in **Fig. 2i-m** following euthanasia at day 56 post-doxycycline treatment, with tumour weight quantified by the subtraction of right kidney mass from left kidney mass. n=9 mice (LacZ and CBF-β sg2 groups), n=10 mice (CBF-β sg1 group). Mean ± SD. One-way ANOVA.

Source data are provided as a Source Data file.

Supplementary Fig. 4

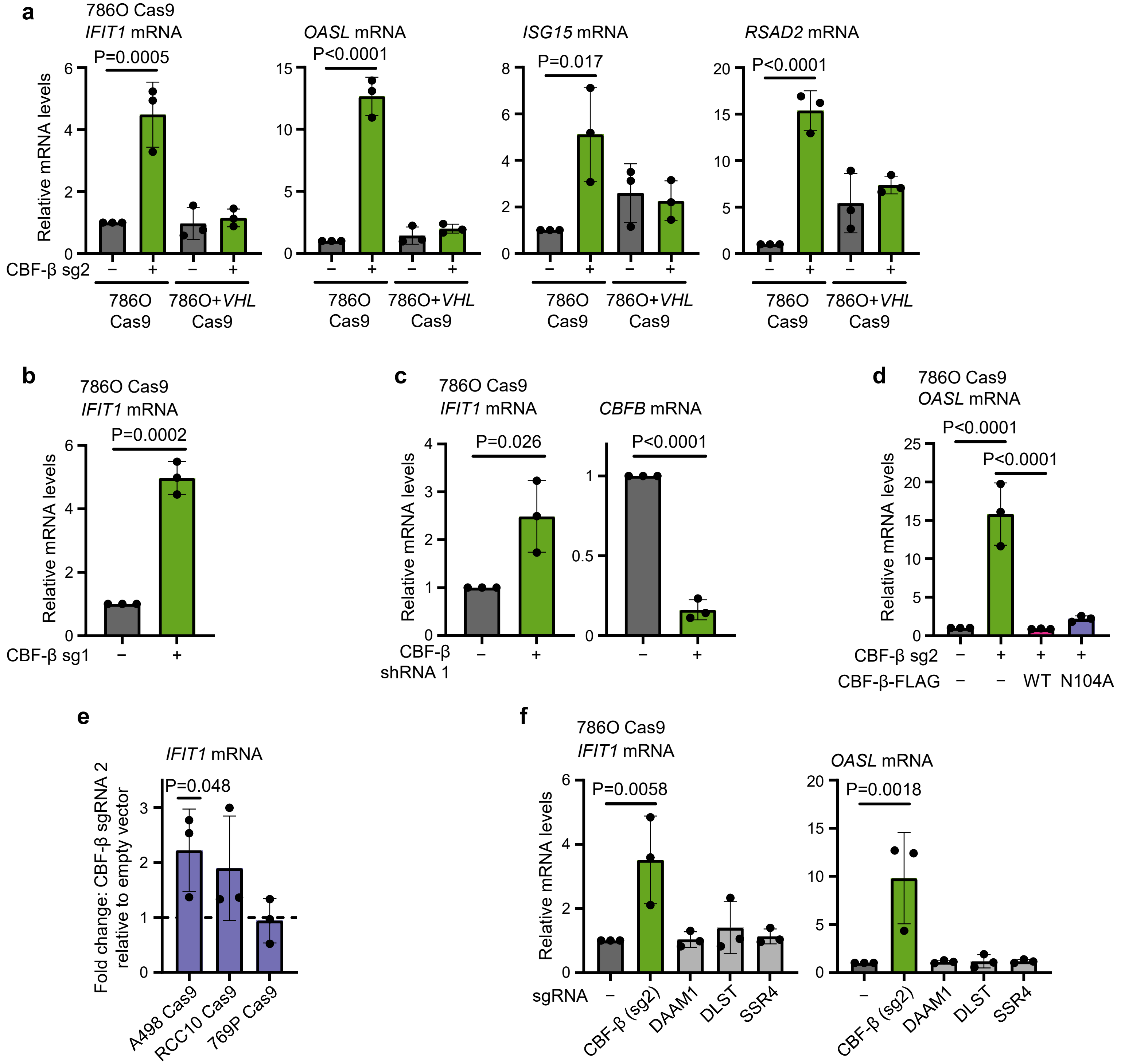

Supplementary Fig. 4. Specific induction of ISG transcription upon *CBFB* knockout

(**a**) qPCR validation of changes in ISG expression in 786O Cas9 and 786O+*VHL* Cas9 cells upon transduction with CBF-β sg2 or an empty vector control. n=3 biologically independent replicates. Mean ± SD. Two-way ANOVA.

(**b,c**) qPCR analysis of changes in *IFIT1* expression in 786O Cas9 cells upon transduction with CBF-β sgRNA 1 (sg1) (**b**) or CBF-β shRNA 1 (**c**), relative to an appropriate empty vector or scrambled shRNA control. n=3 biologically independent replicates. Mean ± SD. Unpaired *t*-test.

(**d**) qPCR analysis of *OASL* expression in 786O Cas9 cells upon transduction with CBF-β sg2 and overexpression vectors encoding CBF-β-FLAG (WT) or CBF-β-FLAG (N104A), or an empty vector control. n=3 biologically independent replicates. Mean ± SD. One-way ANOVA.

(**e**) Expression of *IFIT1* assayed by qPCR in A498, RCC10 and 769P ccRCC cell lines upon transduction with CBF-β sg2, normalised to equivalent cells transduced with an empty vector. n=3 biologically independent replicates. Mean ± SD. Unpaired *t*-test.

(**f**) qPCR analysis of ISGs upon transduction with sgRNAs targeting CBF-β, DAAM1, DLST, or SSR4, relative to an empty vector-transduced control. n=3 biologically independent replicates. Mean ± SD. One-way ANOVA.

Source data are provided as a Source Data file.

Supplementary Fig. 5

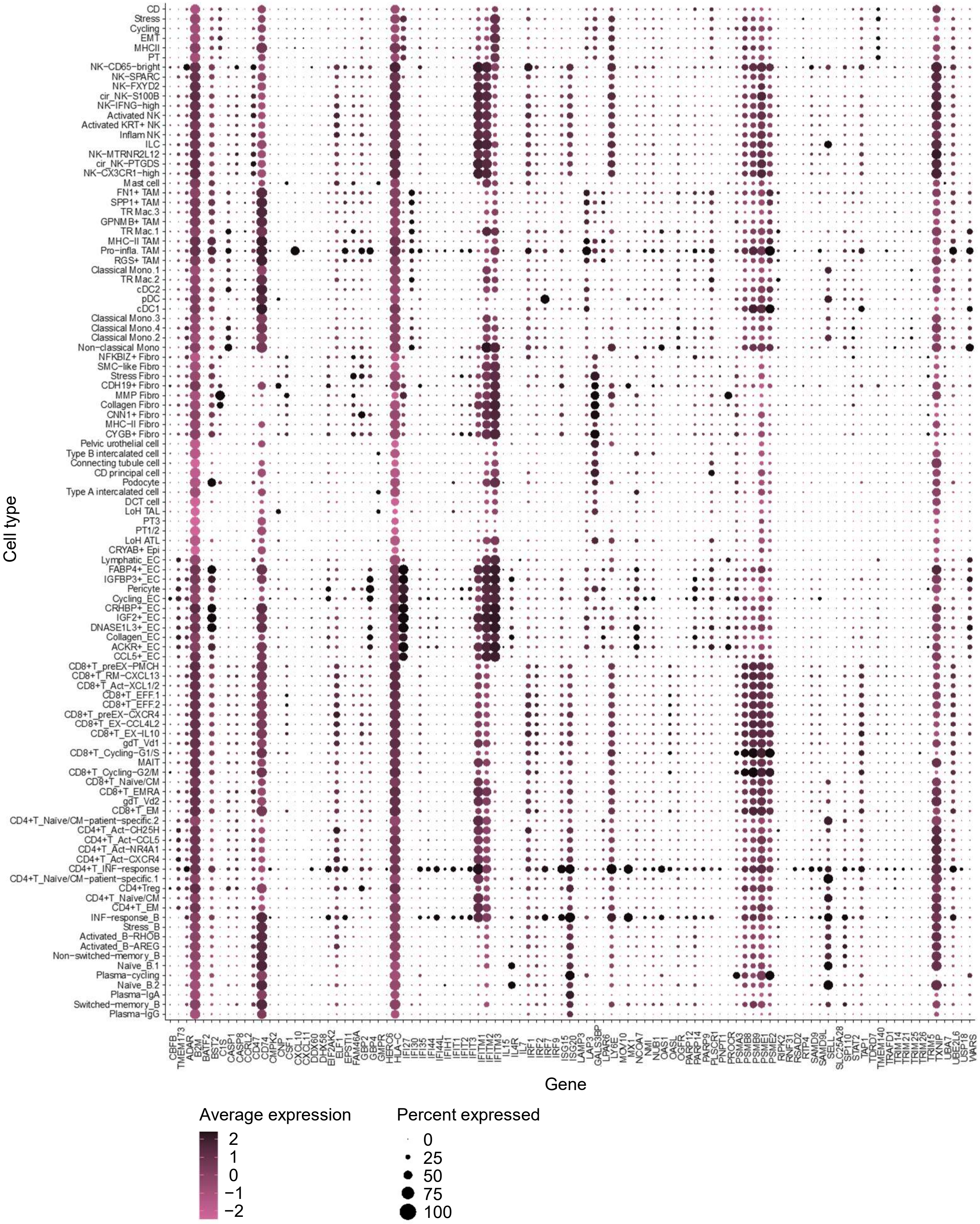

Supplementary Fig. 5. scRNA-Seq analysis of type I IFN genes in ccRCC

Extended analysis of single cell transcriptomic data from patients with ccRCC[2]. The average expression and the percentage of cells that express type I interferon genes is shown for the principal cell types within the tumour micro-environment.

Supplementary Fig. 6

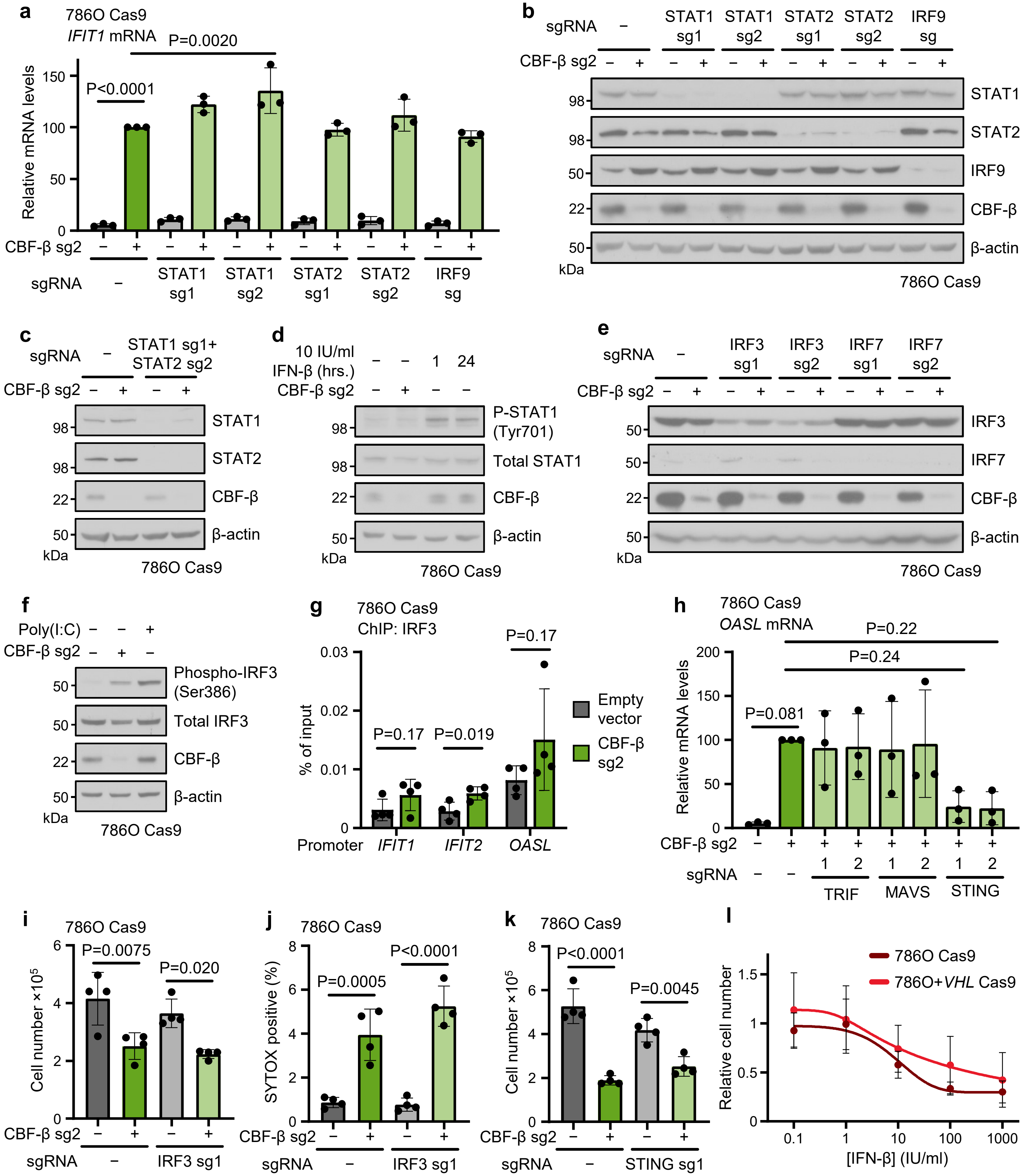

Supplementary Fig. 6. ISG transcription following *CBFB* deletion is mediated by the STING-TBK1-IRF3 axis

(**a,b**) 786O Cas9 cells were transduced with CBF-β sg2 and sgRNAs targeting STAT1, STAT2 or IRF9, or an empty vector. Cells were analysed by qPCR (**a**) or immunoblot (**b**). n=3 biologically independent replicates for (**a**) and (**b**). Mean ± SD. Two-way ANOVA.

(**c**) Immunoblot of 786O Cas9 cells transduced with CBF-β sg2, a vector encoding sgRNAs targeting both STAT1 (sg1) and STAT2 (sg2), or an empty vector control. Representative of 3 biologically independent replicates.

(**d**) CBF-β does not affect the phosphorylation status of STAT1. Cells were transduced with CBF-β sg2 or an empty vector control. Positive controls were additionally treated with 10 IU/ml IFN-β for 1 or 24 hours. Immunoblot representative of 3 biologically independent replicates.

(**e**) Immunoblot of experimental conditions assayed in **Fig. 5c**. Representative of 3 biologically independent replicates.

(**f**) Cells were transduced with CBF-β sg2 or an empty vector control. IRF3 phosphorylation was elicited in positive control cells treated with 20 µg/ml poly(I:C) for 6 hours. Immunoblot representative of 3 biologically independent replicates.

(**g**) Chromatin binding of IRF3 at ISREs within the promoter regions of canonical ISGs. ChIP-qPCR analysis of 786O Cas9 cells transduced with CBF-β sg2 or an empty vector. n=4 biologically independent replicates. Mean ± SD. Unpaired *t*-test.

(**h**) qPCR analysis of *OASL* expression in conditions described in **Fig. 5f**. n=3 biologically independent replicates. Mean ± SD. One-way ANOVA.

(**i-k**) 786O Cas9 cells were transduced with sgRNAs targeting CBF-β (sg2), IRF3 (sg1), or STING (sg1), or an empty vector control, and assayed by proliferation assay (**i**,**k**), or flow cytometry with SYTOX AADvanced dead cell stain (**j**). n=4 biologically independent replicates. Mean ± SD. Two-way ANOVA.

(**l**) Dose-response relationship between IFN-β treatment and cell proliferation over 72 hours in 786O Cas9 and 786O+*VHL* Cas9 cells. n=6 biologically independent replicates. Mean ± SD.

Source data are provided as a Source Data file.

Supplementary Fig. 7

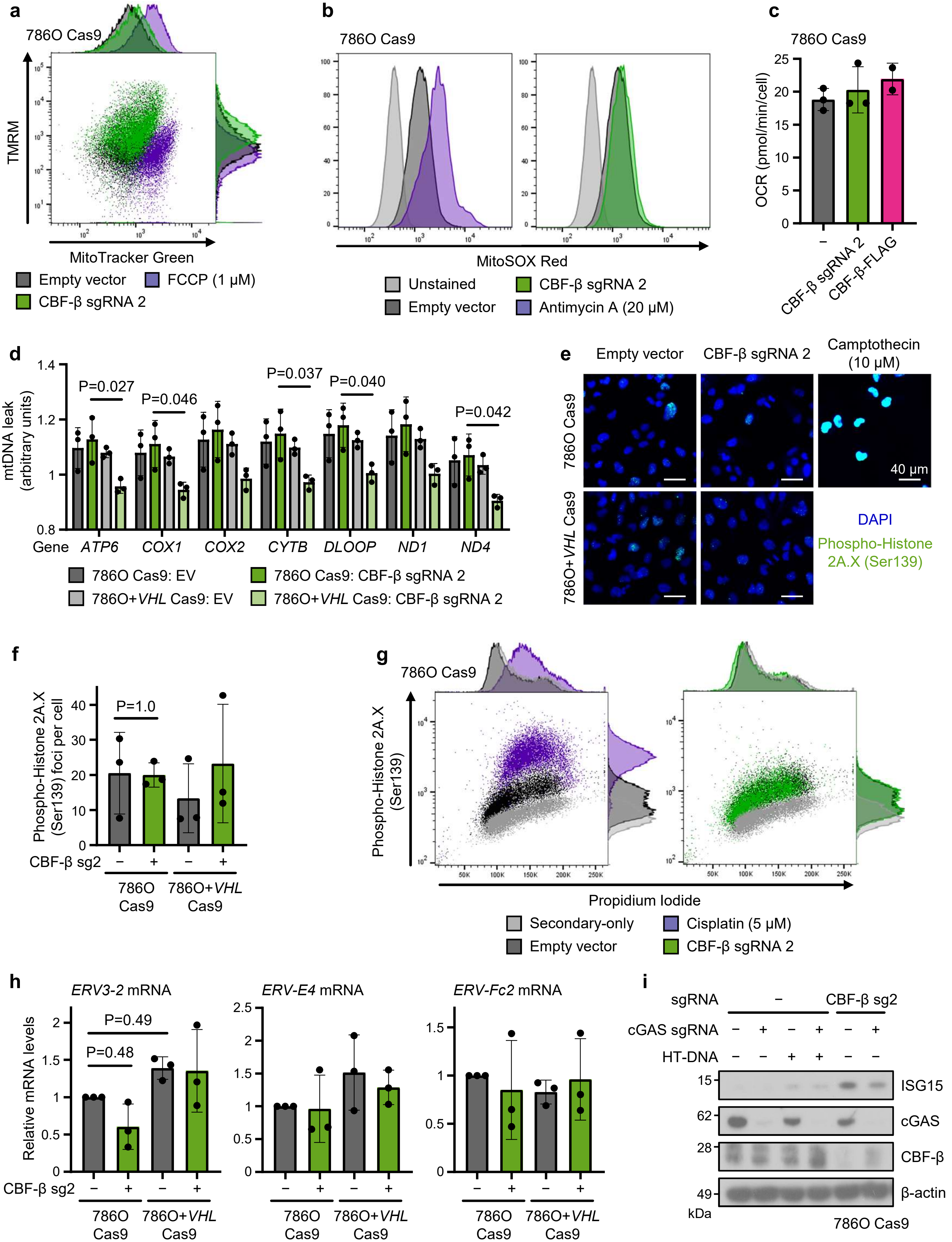

Supplementary Fig. 7. Absence of overt mitochondrial dysfunction or genomic DNA damage following CBF-β loss

(**a**,**b**) 786O Cas9 cells were transduced with CBF-β sg2 or an empty vector and analysed by flow cytometry with TMRM and MitoTracker Green FM (**a**) or MitoSOX Red (**b**) staining to assay mitochondrial membrane potential, total mass, and superoxide generation. Controls were treated for 30 minutes prior to staining with inhibitors of oxidative phosphorylation (1 μM FCCP or 20 μM Antimycin A). Representative of 3 (**a**) or 4 (**b**) biologically independent replicates.

(**c**) 786O Cas9 cells with CBF-β depleted or overexpressed analysed by Mito Stress Test to identify the basal level of respiration, compared to cells transduced with an empty lentiviral vector. OCR: oxygen consumption rate. n=2 (CBF-β-FLAG) or 3 (control and CBF-β sg2) biologically independent replicates. Mean ± SD.

(**d**) Leakage of mitochondrial DNA (mtDNA) from cells transduced with CBF-β sg2 or an empty vector. The abundance of mitochondrial genes within the cytoplasm was determined by qPCR and normalised to the quantity of nuclear *ACTB* DNA for each sample. n=3 biologically independent replicates. Mean ± SD. Two-way ANOVA.

(**e**,**f**) Foci of histone 2A.X Ser139 phosphorylation (γ-H2A.X) identified by confocal microscopy in cells transduced with CBF-β sg2 or an empty vector. Controls were treated with 10 μM Camptothecin for 24 hours before analysis. Representative image (**e**), and quantified number of foci per cell (**f**). 40 μm scale bar. Each point represents the mean of approximately 50 cells acquired and analysed together per biological replicate. n=3 biologically independent replicates. Mean ± SD. Two-way ANOVA.

(**g**) Flow cytometry analysis of γ-H2A.X. Cells were transduced with CBF-β sg2 or an empty vector, and a population of empty vector-transduced controls treated with 5 μM Cisplatin 24 hours prior to analysis. Representative of 4 biologically independent replicates.

(**h**) qPCR analysis of *ERV3-2*, *ERV-E4* and *ERV-Fc2* expression in 786O Cas9 and 786O+*VHL* Cas9 cells upon transduction with CBF-β sg2 or an empty vector control. n=3 biologically independent replicates. Mean ± SD. Two-way ANOVA.

(**i**) Immunoblot of 786O Cas9 cells as described in **Fig. 6e**, untransfected or following transfection with 0.5 μg/ml HT-DNA for 6 hours. Representative of 4 biologically independent replicates.

Source data are provided as a Source Data file.

Supplementary Fig. 8

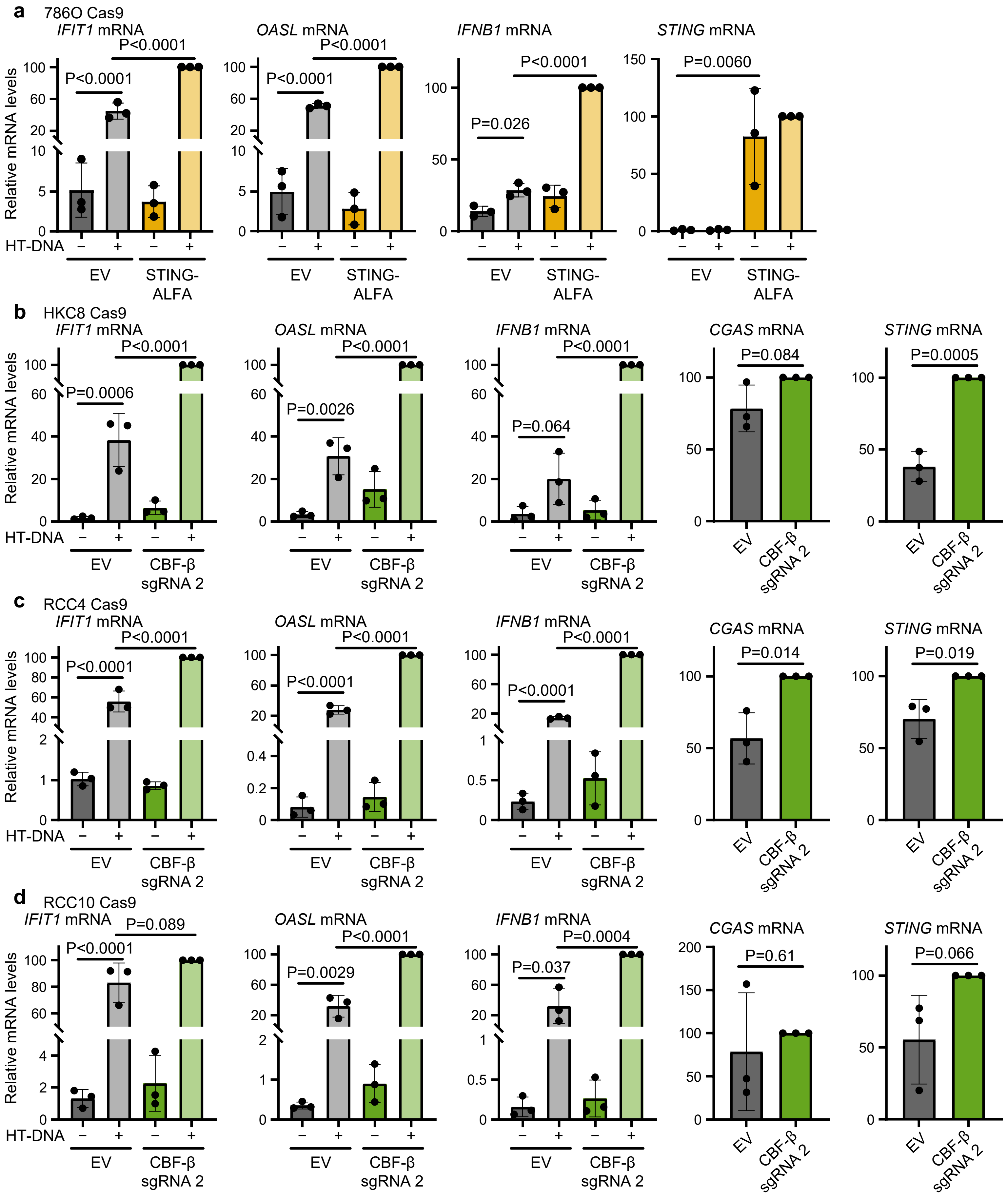

Supplementary Fig. 8. Repression of STING by CBF-β/RUNX across kidney cell lines

(**a**) ISG transcription in response to exogenous DNA transfection is sensitised by STING overexpression. 786O Cas9 cells were transduced with vectors encoding ALFA-tagged STING or an empty vector control, treated with 1 μg/ml doxycycline for 24 hours and either transfected with 0.5 μg/ml HT-DNA for the final 6 hours or left untransfected. qPCR analysis of *IFIT1*, *OASL*, *IFNB1* and *STING* mRNA expression. n=3 biologically independent replicates. Mean ± SD. Two-way ANOVA.

(**b-d**) The expression of *STING* and downstream ISGs is induced by *CBFB* knockout in renal proximal tubule epithelial (HKC8), and ccRCC (RCC4 and RCC10) cell lines. qPCR analysis of HKC8 Cas9 (**b**), RCC4 Cas9 (**c**), and RCC10 Cas9 (**d**) cells transduced with CBF-β sg2 or an empty vector (EV), and either transfected with 0.5 μg/ml HT-DNA for 6 hours or left untransfected. n=3 biologically independent replicates. Mean ± SD. Two-way ANOVA (*IFIT1*, *OASL* and *IFNB1*), or unpaired *t*-test (*CGAS* and *STING*).

Source data are provided as a Source Data file.

Supplementary Fig. 9

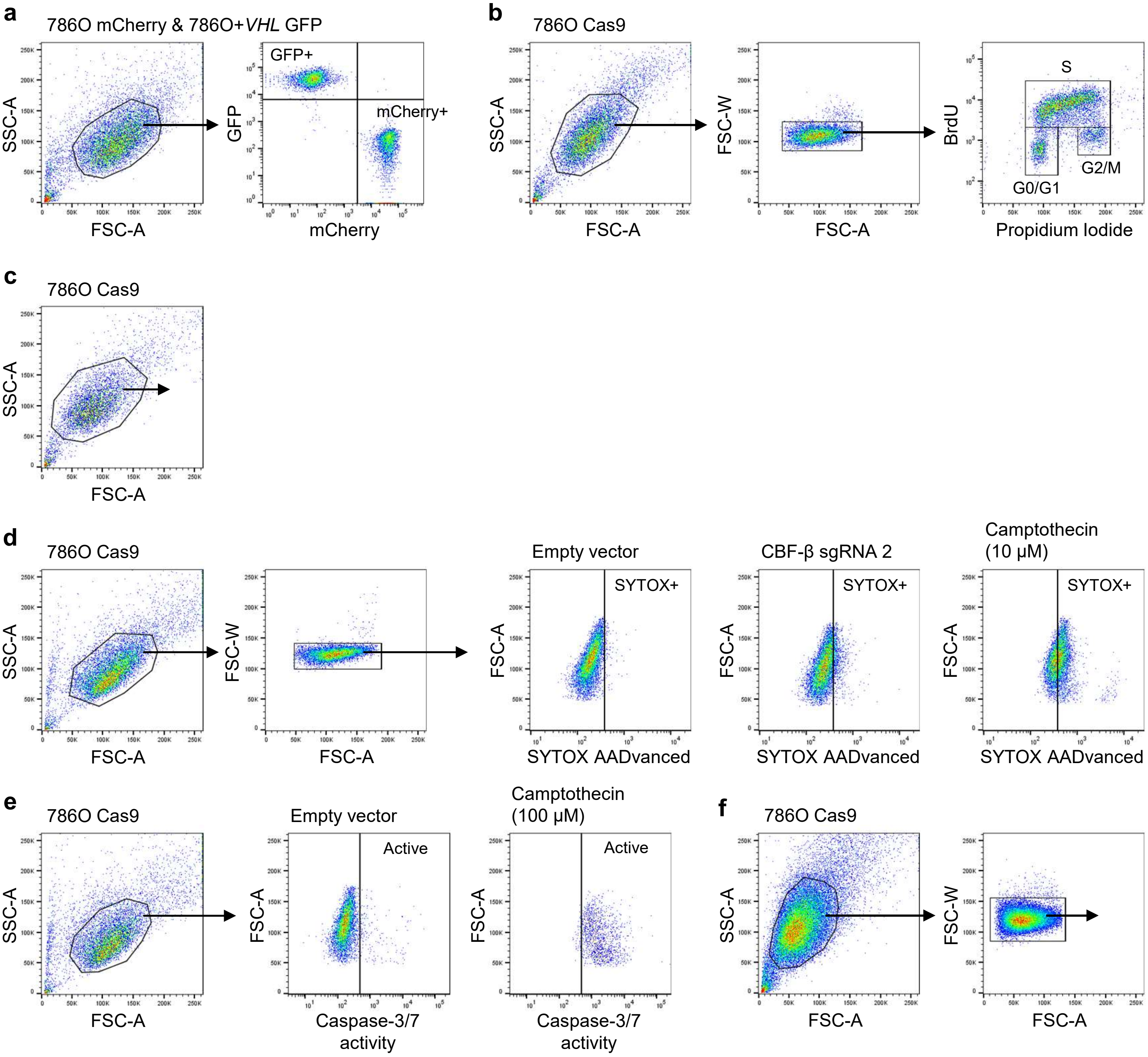

Supplementary Fig. 9. Gating strategies for flow cytometry

(**a-f**) Representative gating strategies used in flow cytometry experiments in 786O Cas9 cells: competitive growth assay in **Fig. 1f,g** and **Fig. 3a,f** (**a**); cell cycle analysis with BrdU and PI in **Fig. 2e** (**b**); cell cycle analysis with PI alone in **Supplementary Fig. 3a** (**c**); SYTOX cell death assays in **Fig. 2f**, **Supplementary Fig. 3d** and **Supplementary Fig. 6j** (**d**); Caspase-3/7 Green apoptosis assays in **Supplementary Fig. 3b** (**e**); and staining of γ-H2A.X, and for functional mitochondrial assays in **Supplementary Fig. 7a,b,g** (**f**). SSC-A: side scatter (area). FSC-A: forward scatter (area). FSC-W: forward scatter (width).

Supplementary Table 1. Details of cell lines, antibodies, reagents, plasmids and software

Cell lines

Cell line	Source	Identifier
A498	P. Schraml lab	N/A
HEK293T	ATCC	Cat#CRL-3216
HKC8	P.H. Maxwell lab	N/A
HK2	ATCC	Cat#CRL-2190
RCC4	P.H. Maxwell lab	N/A
RCC4+*VHL*	P.J. Ratcliffe lab	N/A
RCC10	P.H. Maxwell lab	N/A
786O	ATCC	Cat#CRL-1932
786O+*VHL*	W.G. Kaelin Jr. lab	N/A
769P	P. Schraml lab	N/A

Antibodies

Antibody	Source	Identifier
Rat anti-BrdU	Abcam	Cat#ab6326
Rabbit anti-CBF-β	Cell Signaling Technology	Cat#62184
Rabbit anti-CBF-β	Diagenode	Cat#C15310002
Rabbit anti-cGAS	Cell Signaling Technology	Cat#D1D3G
Mouse anti-FLAG M2	Sigma	Cat#F1804
Mouse anti-GFP	Roche	Cat#11814460001
Rabbit anti-H3K36me3	Cell Signaling Technology	Cat#9763
Mouse anti-HIF-1α	BD Biosciences	Cat#610959
Mouse anti-HIF-1β	Cell Signaling Technology	Cat#5537S
Rabbit anti-IFI44	ThermoFisher	Cat#PA5-65370
Mouse anti-IRF3	BioLegend	Cat#655704
Rabbit anti-IRF3	Cell Signaling Technology	Cat#11904
Rabbit anti-IRF7	Cell Signaling Technology	Cat#13014
Rabbit anti-IRF9	Cell Signaling Technology	Cat#76684
Rabbit anti-ISG15	Santa Cruz	Cat#50366
Rabbit anti-KEAP1	Cell Signaling Technology	Cat#4678
Mouse anti-MDA5	Hertzog et al.[3]	N/A
Rabbit anti-MX1	Cell Signaling Technology	Cat#37849
Rabbit anti-NRF2	Cell Signaling Technology	Cat#12721
Rabbit anti-PARP1	Cell Signaling Technology	Cat#9542S
Rabbit anti-Phospho-Histone 2A.X (Ser139)	Cell Signaling Technology	Cat#9718
Rabbit anti-Phospho-IRF3 (Ser386)	Cell Signaling Technology	Cat#37829
Rabbit anti-Phospho-STAT1 (Tyr701)	Cell Signaling Technology	Cat#9167
Rabbit anti-RUNX1	Cell Signaling Technology	Cat#4344
Rabbit anti-RUNX2	Cell Signaling Technology	Cat#8486
Rabbit anti-STING	Cell Signaling Technology	Cat#13647
Goat anti-Rabbit Alexa-Fluor 488 IgG	Invitrogen	Cat#A11034
Goat anti-Rabbit Alexa-Fluor 647 IgG	Invitrogen	Cat#A21245
Goat anti-Rat Alexa-Fluor 647 IgG	Invitrogen	Cat#A21247
Goat anti-Mouse Peroxidase-AffiniPure IgG	Jackson	Cat#115-035-146
Goat anti-Rabbit Peroxidase-AffiniPure IgG	Jackson	Cat#111-035-045

Reagents

Reagent	Source	Identifier
AF1 plus DAPI	CitiFluor	Cat#17970-125
Ampicillin	ThermoFisher	Cat#J6380706
Antimycin A	Alfa Aesar	Cat#J63522
Azido-PEG3-Azide	Lumiprobe	Cat#207
Bafilomycin A1	Alfa Aesar	Cat#J61835
BAY 11-7085	MedChemExpress	Cat#HY-10257
Benzonase	Sigma	Cat#E1014
Blasticidin	TOKU-E	Cat#B001
Bromodeoxyuridine (BrdU)	Abcam	Cat#ab142567
Camptothecin	Cayman Chemical	Cat#11694
CellEvent Caspase-3/7 Green detection reagent	ThermoFisher	Cat#C10423
Cisplatin	Sigma	Cat#232120
cOmplete EDTA-free protease inhibitor cocktail	Roche	Cat#46931590001
Crystal violet	PhytoTech	Cat#C1830
DMEM	Sigma	Cat#D6429
DMSO	Santa Cruz	Cat#sc359032
Doxycycline	Cayman Chemical	Cat#14422
D-PBS	ThermoFisher	Cat#J67802.K2
FCCP	Cayman Chemical	Cat#15218
FCS	Sigma	Cat#F0392
Ferrostatin-1	Abcam	Cat#ab146169
GSK8612	Selleck Chemicals	Cat#S8872
HBSS	Gibco	Cat#14175095
Herring Testes DNA (HT-DNA)	Sigma	Cat#D6898
Hexylene glycol	Sigma	Cat#112100
Hoescht 33342	Tocris Bioscience	Cat#5117/50
Hygromycin	ThermoFisher	Cat#10687010
IFN-β	PeproTech	Cat#300-02BC
Lipofectamine 2000	Invitrogen	Cat#11668027
Matrigel	Corning	Cat#354234
MitoSOX Red	ThermoFisher	Cat#M36008
MitoTracker Green FM	ThermoFisher	Cat#M7514
Necrostatin-1s	Cayman Chemical	Cat#20924
Pierce Enhanced Chemiluminescent Substrate	ThermoFisher	Cat#32209
Pierce Protein G magnetic beads	ThermoFisher	Cat#88848
Poly(I:C)	ApexBio	Cat#B5551
Propidium iodide	Sigma	Cat#P4864
Proteinase K	Thermo Scientific	Cat#EO0491
Protoscript II Reverse Transcriptase	NEB	Cat#M0368X
PureLink DNase	ThermoFisher	Cat#12185010
Purina rodent chow #5001 with 2000 ppm doxycycline	Research Diets	Cat#C11300-2000i
Puromycin	MP Biomedicals	Cat#200-387-8
PVDF membranes	Merck	Cat#IPVH00010
RNase A	Thermo Scientific	Cat#EN0531
RNase H	Thermo Scientific	Cat#EN0202
RPMI-1640	Sigma	Cat#R8758
Seahorse XF RPMI medium, pH 7.4	Agilent	Cat#103576-100
SeeBlue Plus2	Invitrogen	Cat#LC5925
SuperSignal West Dura Extended Duration Substrate	ThermoFisher	Cat#34075

Reagents (continued)

Reagent	Source	Identifier
SuperSignal West Pico Plus Chemiluminescent Substrate	ThermoFisher	Cat#34577
SYBR Green PCR Master Mix	ThermoFisher	Cat#4309155
SYTOX AADvanced	ThermoFisher	Cat#S10349
Thymidine	Sigma	Cat#T1895
TMRM	ThermoFisher	Cat#T668
TMTpro labels	ThermoFisher	Cat#A44520
Trypsin/lysC mix	Promega	Cat#V5071
UltraPure™ Phenol:Chloroform:Isoamyl Alcohol (25:24:1, v/v)	Invitrogen	Cat#15593031
VRT-043198	MedChemExpress	Cat#HY-112226

Plasmids

Plasmid	Source	Identifier
Lenti-Cas9-2A-Blast	Hart et al.[4]	Cat#Addgene-73310
pC.SIREN.Puro	Schaller et al.[5]	N/A
pCFD5	Port and Bullock[6]	Cat#Addgene-73914
pCMVR8.91 (Lentiviral Gag/Pol)	Demaison et al.[7]	N/A
pCW57.1	D.E. Root lab	Cat#Addgene-41393
pCW57.1-STING-ALFA	This paper	N/A
pDonor-Clover- LoxP-Puro-STING	P.J. Lehner lab	N/A
pHRSIN-pSFFV-GFP	Dupont et al.[8]	N/A
pHRSIN-pSFFV-CBF-β (N104A)-FLAG-Puro	This paper	N/A
pHRSIN-pSFFV-CBF-β (WT)-FLAG-Puro	This paper	N/A
pHRSIN-pSFFV-mCherry-Puro	This paper	N/A
pHRSIN-pSFFV-RUNX1-GFP-Puro	This paper	N/A
pHRSIN-pSFFV-RUNX2-Puro	This paper	N/A
pHRSIN-pSFFV-pPGK-Puro	Demaison et al.[7]	N/A
pHRSIN-SFFV-eGFP-P2A-Vif	Marelli et al.[9]	N/A
pHRSIN-pSFFV-luciferase-Puro	P.J. Lehner lab	N/A
pKLV-U6gRNA(BbsI)-PGKblast2ABFP	P.J. Lehner lab	N/A
pKLV-U6gRNA(BbsI)-PGKpuro2ABFP	Koike-Yusa et al.[10]	Cat#Addgene-50946
pLCKO2-TKOv3	Mair et al.[11]	Cat#Addgene-125517
pMD.G (Lentiviral VSVG)	Demaison et al.[7]	N/A
pPB-cT3G-cERP2-RUNX1	Pierson Smela et al.[12]	Cat#Addgene-192928
pPB-cT3G-cERP2-RUNX2	Pierson Smela et al.[12]	Cat#Addgene-192926
pSpCas9(BB)-T2A-Puro	Ran et al.[13]	Cat#Addgene-48139
tet-pLKO-sgRNA-puro	Huang et al.[14]	Cat#Addgene-104321

Software

Software	Source	Identifier
BAGEL2	Kim and Hart[15]	https://github.com/hart-lab/bagel
BEDTools v2.31.1	Quinlan and Hall[16]	https://github.com/arq5x/bedtools2
Bowtie 2 v2.5.3	Langmead and Salzberg[17]	https://bowtie-bio.sourceforge.net/bowtie2/index.shtml
ChIPseeker v1.44.0	Yu et al.[18]	https://www.bioconductor.org/packages/release/bioc/html/ChIPseeker.html
Cutadapt v4.4	Martin[19]	RRID:SCR_011841
deepTools2	Ramírez et al.[20]	https://usegalaxy.eu
DESeq2	Love et al.[21]	RRID:SCR_015687
ENCODE	The ENCODE Project Consortium[22]	https://www.encodeproject.org
FACSDiva v8.0.3	BD Biosciences	RRID: SCR_001456
FastQC v0.12.1	Babraham Bioinformatics	https://github.com/s-andrews/FastQC
fgsea v1.20.0	Korotkevich et al.[23]	RRID:SCR_020938
Fiji (ImageJ v1.54p)	Schindelin et al.[24]	https://fiji.sc
FlowJo v10	BD Biosciences	RRID:SCR_008520
HISAT2 v2.2.1	Kim et al.[25]	RRID:SCR_015530
IGV v2.16.0	Robinson et al.[26]	RRID:SCR_011793
limma v3.15	Ritchie et al.[27]	RRID:SCR_010943
MACS2 v2.2.9.1	Zhang et al.[28]	http://liulab.dfci.harvard.edu/MACS
MultiQC v1.21	Ewels et al.[29]	https://seqera.io/multiqc
Peaks 11	Bioinfor	https://www.bioinfor.com/peaks-11
Picard v3.1.1	Broad Institute	RRID:SCR_006525
Prism v9.5.1	GraphPad	RRID:SCR_002798
Python v3.10	Python Software Foundation	RRID:SCR_008394
R v4.1.2	R Core Team	RRID:SCR_001905
Salmon	Patro et al.[30]	RRID:SCR_017036
SAMtools v1.20	Li et al.[31]	https://samtools.sourceforge.net
sceasy v0.0.7	Cakir et al.[32]	https://github.com/cellgeni/sceasy
seurat v5.1.0	Hao et al.[33]	https://satijalab.org/seurat
Snakemake v8.25.5	Mölder et al.[34]	https://snakemake.github.io
STAR v2.7.10b	Dobin et al.[35]	RRID:SCR_004463
survminer v0.4.9	Kassambara et al.[36]	RRID:SCR_021094
TEtranscripts v2.2.3	Jin et al.[37]	RRID:SCR_023208
TrimGalore	Krueger et al.	https://zenodo.org/records/7598955
WiggleTools v1.2.11	Zerbino et al.[38]	https://github.com/Ensembl/Wiggletools
UCSC Genome Browser	Perez et al.[39]	https://genome.ucsc.edu

Supplementary Table 2. Oligonucleotide sequences

CBF-β gBlock sequence

GTATTTTGCAGAGCTGGCGAATGCCGCGCGTCGTGCCCGACCAGAGAAGCAAGTTCGAGAACGAGGAGTTT
TTTAGGAAGCTGAGCCGCGAGTGTGAGATTAAGTACACGGGCTTCAGGGACCGGCCCCACGAGGAACGCC
AGGCACGCTTCCAGAACGCCTGCCGCGACGGCCGCTCGGAAATCGCTTTTGTGGCCACAGGAACCAATCTG
TCTCTCCAGTTTTTTCCGGCCAGCTGGCAGGGAGAACAGCGACAAACACCTAGCCGAGAGTATGTCGACTTA
GAAAGAGAAGCAGGCAAGGTATATTTGAAGGCTCCCATGATTCTGAATGGAGTCTGTGTTATCTGGAAAGGC
TGGATTGATCTCCAAAGACTGGATGGTATGGGCTGTCTGGAGTTTGATGAGGAGCGAGCCCAGCAGGAGGA
TGCATTAGCACAACAGGCCTTTGAAGAGGCTCGGAGAAGGACACGCGAATTTGAAGATAGAGACAGGTCTC
ATCGGGAGGAAATGGAGGCAAGAAGACAACAAGACCCTAGTCCTGGTTCCAATTTAGGTGGTGGTGATGAC
CTCAAACTTCGTTAAACGCGTTGAACACTTCACAG

Primers used to clone gene expression vectors

Experiment	Primer	Sequence
pHRSIN-pSFFV-CBF-β (N104A)-FLAG-Puro pHRSIN-pSFFV-CBF-β (WT)-FLAG-Puro	Fw	CAGTCCTCCGACAGACTGAGTCGCCCGGGGGGATCC GCCACCATGCCGCGCGTCGTGCCCGACCAGAGAAGC
	Rv	CCGTCATGGTCTTTGTAGTCAGCCCGCTCGAGCGG CCGCCCACGAAGTTTGAGGTCATCACCACCACCTAA
	N104A mutation (Fw)	CTCCCATGATTCTGGCTGGAGTCTGTGTTATC
	N104A mutation (Rv)	GATAACACAGACTCCAGCCAGAATCATGGGAG
To clone RUNX1-GFP into pHRSIN-pSFFV-Puro	Fw	GTCCTCCGACAGACTGAGTCGCCCGGGGGGATCC GCCACCATGGCTTCAGACAGCATATTTGAGTCATTTC
	Rv	CTCACCGCGGTGGCGACCGGTGGATCTGCACG CGTGTAGGGCCTCCACACGGCCTCCTCCAG
To clone RUNX2 into pHRSIN-pSFFV-Puro	Fw	GTCCTCCGACAGACTGAGTCGCCCGGGGGGATCC GCCACCATGGCATCAAACAGCCTCTTCAGCACAGTG
	Rv	CTCACCGCGGTGGCGACCGGTGGATCTGCACG CGTTATGGTCGCCAAACAGATTCATCCATTCTG
To clone mCherry into pHRSIN-SFFV	Fw	CAGTCCTCCGACAGACTGAGTCGCCCGGGGGGAT CCGCCACCATGGTGAGCAAGGGCGAGGAGGATAAC
	Rv	CTTGCATGCCTGCAGGTCGACTCTAGAGTCGCG GCCGCTCACTTGTACAGCTCGTCCATGCCGCCGG
To clone STING-ALFA into pCW57.1	Fw	GTGAACCGTCAGATCGCCTGGAGAATTGGCTAGC ATCAATGCCCCACTCCAGCCTGCATCCATCCATC
	Rv (step 1)	TTACTCGGTCAGTCTTCTTCTCAGCTCCTCCTCCAG TCTGCTACCGGTGAATTCAGAGAAATCCGTGCGG AGAGGGAGGGGC
	Rv (step 2)	CAACCCCGGATCCTTAGTGGTGGTGGTGGTGG TGGTTACTCGGTCAGTCTTCTTCTCAGCTCCTC

sgRNA sequences

Target	sgRNA sequence
CBF-β (sg1)	GAAGCTGAGCCGCGAGTGTG
CBF-β (sg2)	GCCTTGCAGATTAAGTACAC
cGAS	AAATTAAGAAGAAACATGG
DAAM	CAGCCGATACGTGATTTCT
DLST	TACACAACCTTCCTGCTGTT
HIF1β	CAGTCCTCCGTCTCCTCACC
IRF3 (sg1)	AACCAGAGGGCATAGCG
IRF3 (sg2)	ATCTGATTACCTTCACGGA
IRF7 (sg1)	ATGCTGCGGGATAACTCGG
IRF7 (sg2)	AAGCAGCTGCGCTACACGG
IRF9	GGCTCAGCAACATCCATG
KEAP1 (sg1)	GTTACGGGGCACGCTCATGG
KEAP1 (sg2)	AGCACCGGCGAAGTGCCCTG
LacZ	CAGCTGGCGTAATAGCGAAG
MAVS (sg1)	TGTTCACAGGCATCAAGG
MAVS (sg2)	ACGGGAGCAGCAGAAATG
NRF2 (sg1)	CATTAATTCGGGATATACGT
NRF2 (sg2)	GGACATTGAGCAAGTTTGGG
RUNX1	GCTCCGTGCTGCCTACGCAC
RUNX2	GTAGGTGTGGTAGTGAGTGG
RUNX3	GGACGTGCCGGATGGTACGG
SSR4	TGAGGTTAGATTCTTCGACG
STAT1 (sg1)	ACGTTGGAGATCACCACAA
STAT1 (sg2)	AGGTCATGAAAACGGATGG
STAT2 (sg1)	TTGGCTGGCCAGAACACCG
STAT2 (sg2)	GGCCCAGCAAGCTCCAGG
STING (sg1)	CACCCCACAGTCCAATGGG
STING (sg2)	AAAAAGGGAATTTCAACG
TRIF (sg1)	AAGCTGGGCCAGGAAACTG
TRIF (sg2)	TGCGTGGTGGATAATGAG

shRNA sequences

Target	shRNA sequence
CBF-β (sh1)	TGACCTCAAACTTCGTTAATT
CBF-β (sh2)	GAGAAGCAGGCAAGGTATATT
CBF-β (sh3)	CCGCGAGTGTGAGATTAAGTA
Scrambled sh	GCATAATTAATATCCGCGTGT

qPCR primers

Target	Forward primer	Reverse primer
ATP6	TCCCTCTACACTTATCATCTTCAC	GACAGCGATTTCTAGGATAGTC
CBFB	GCTCGGAAATCGCTTTTGTGG	CGGCTAGGTGTTTGTCGCT
CGAS	CGGGAGCTACTATGAGCACG	GCCATGTTTCTTCTTGGAAACCA
COX1	ATATTTCACCTCCGCTACCA	TCAGCTAAATACTTTGACGCC
COX2	ACGCATCCTTTACATAACAGAC	GCCAATTGATTTGATGGTAAGG
CYTB	ATCACTTTATTGACTCCTAGCC	TGGTTGTCCTCCGATTCAG
DLOOP	AGCACATTACAGTCAAATCCCTTCTC	CACGGAGGATGGTGGTCAAG
ERV-E4	GGAGTAATAACAGTATTAGGAACCTGCT	CTTGTGCTGAACTATTTTGGTGAATT
ERV-Fc2	CTCCATTAGTAGCAGTTCCTCTCC	GAGAATAGTGGGACCTGTCCTTT
ERV3-2	AGCCATTTACAAAGAAAGGGGAC	CTATGCCGCCTCTTGTCTGAT
GLUT1	CCAGGGTAGCTGCTGGAGC	TGGCATGGCGGGTTGT
HMOX1	CAACATCCAGCTCTTTGAGG	GGCAGAATCTTGCACTTTG
IFIT1 (qPCR)	GGAATACACAACCTACTAGCC	CCAGGTCACCAGACTCCTCA
IFIT1 (ChIP)	AAGCAAAACCCTGCAGAACG	AGACATTATGGCAGAGGAGCAA
IFIT2 (ChIP)	AAGAGGGCCAGCTCCATTTT	TCAGGAGGGAACAAACCACC
IFNB1	CAGCATCTGCTGGTTGAAGA	CATTACCTGAAGGCCAAGGA
ISG15	CTCTGAGCATCCTGGTGAGGAA	AAGGTCAGCCAGAACAGGTCGT
ND1	CCCTAAAACCCGCCACATCT	GAGCGATGGTGAGAGCTAAGGT
ND2	CCCTTCCTTGTACTATCCCT	TTTGTCGTAGGCAGATGGAG
NQO1	CCTGCCATTCTGAAAGGCTGGT	GTGGTGATGGAAAGCACTGCCT
OASL (qPCR)	GCGGAGCCCATCACGGTCAC	AGCACCACCGCAGGCCTTGA
OASL (ChIP)	TAGTCCAGTGAGCCCCCAAA	GGGACCCCTGTCCTCCTTAT
PHD3	TCCTGCGGATATTTCCAGAGG	GGTTCCTACGATCTGACCAGAA
RSAD2	CCAGTGCAACTACAAATGCGGC	CGGTCTTGAAGAAATGGCTCTCC
RUNX1	CACTGTGATGGCTGGCAATGATG	CTCTGTGGTAGGTGGCGACTTG
RUNX2	AGCCCTCGGAGAGGTACCA	CGGAGCTCAGCAGAATAATTTTC
RUNX3	GACTGTGATGGCAGGCAATGA	CGAAGCGAAGGTCGTTGAA
STING (qPCR)	CCTGAGTCTCAGAACAACTGCC	GGTCTTCAAGCTGCCCACAGTA
STING (ChIP 1)	TCCACAACACTCTAGCCCTG	GGAAATACCCTCCTTCCCAGC
STING (ChIP 2)	CCAAACCGCAGCTTTACTGG	ATCAGGGCTTTGAGGGAAGG
STING (ChIP 3)	TGGGACATCGTGGAGGTACT	CCCCTATCTCCCTGTTCCAGA
STING (ChIP 4)	GATGTCAAGTCCTGGACCCTTC	GGACTGGGGGCTCTCTTCAG
VEGFA	TACCTCCACCATGCCAAGTG	ATGATTCTGCCCTCCTCCTTC
β-Actin	CTGGGAGTGGGTGGAGGC	TCAACTGGTCTCAAGTCAGTG

Primers used to amplify and sequence the TKOv3 library

Reaction	Primer	Sequence
Outer PCR	Fw	GAGGGCCTATTTCCCATGATTC
	Rv	CAAACCCAGGGCTGCCTTGGAA
Inner PCR	Fw	AATGATACGGCGACCACCGAGATCTACACTCTCTTGTGGAAAGGACGAGGTACCG
	Rv	CAAGCAGAAGACGGCATACGAGATNNNNNNNGTGACTGGAGTTCAGACGTGTGCTC TTCCGATCTATTTTAACTTGCTATTTCTAGCTCTAAAAC (NNNNNNN: Unique barcode sequence for multiplexing)
Illumina Sequencing		ACACTCTCTTGTGGAAAGGACGAAACACCG

Supplementary References

1. Hart, T. *et al.* Evaluation and Design of Genome-Wide CRISPR/SpCas9 Knockout Screens. *G3 Genes Genomes Genet.* **7**, 2719–2727 (2017).
2. Li, R. *et al.* Mapping single-cell transcriptomes in the intra-tumoral and associated territories of kidney cancer. *Cancer Cell* **40**, 1583-1599.e10 (2022).
3. Hertzog, J. *et al.* Infection with a Brazilian isolate of Zika virus generates RIG-I stimulatory RNA and the viral NS5 protein blocks type I IFN induction and signaling. *Eur. J. Immunol.* **48**, 1120–1136 (2018).
4. Hart, T. *et al.* High-Resolution CRISPR Screens Reveal Fitness Genes and Genotype-Specific Cancer Liabilities. *Cell* **163**, 1515–1526 (2015).
5. Schaller, T. *et al.* HIV-1 Capsid-Cyclophilin Interactions Determine Nuclear Import Pathway, Integration Targeting and Replication Efficiency. *PLoS Pathog.* **7**, e1002439 (2011).
6. Port, F. & Bullock, S. L. Augmenting CRISPR applications in Drosophila with tRNA-flanked Cas9 and Cpf1 sgRNAs. *Nat. Methods* **13**, 852–854 (2016).
7. Demaison, C. *et al.* High-Level Transduction and Gene Expression in Hematopoietic Repopulating Cells Using a Human Imunodeficiency Virus Type 1-Based Lentiviral Vector Containing an Internal Spleen Focus Forming Virus Promoter. *Hum. Gene Ther.* **13**, 803–813 (2002).
8. Dupont, L. *et al.* The SMC5/6 complex compacts and silences unintegrated HIV-1 DNA and is antagonized by Vpr. *Cell Host Microbe* **29**, 792-805.e6 (2021).
9. Marelli, S. *et al.* Antagonism of PP2A is an independent and conserved function of HIV-1 Vif and causes cell cycle arrest. *eLife* **9**, e53036 (2020).
10. Koike-Yusa, H., Li, Y., Tan, E.-P., Velasco-Herrera, M. D. C. & Yusa, K. Genome-wide recessive genetic screening in mammalian cells with a lentiviral CRISPR-guide RNA library. *Nat. Biotechnol.* **32**, 267–273 (2014).
11. Mair, B. *et al.* Essential Gene Profiles for Human Pluripotent Stem Cells Identify Uncharacterized Genes and Substrate Dependencies. *Cell Rep.* **27**, 599-615.e12 (2019).
12. Pierson Smela, M. D. *et al.* Directed differentiation of human iPSCs to functional ovarian granulosa-like cells via transcription factor overexpression. *eLife* **12**, e83291 (2023).
13. Ran, F. A. *et al.* Genome engineering using the CRISPR-Cas9 system. *Nat. Protoc.* **8**, 2281–2308 (2013).
14. Huang, H.-T. *et al.* MELK is not necessary for the proliferation of basal-like breast cancer cells. *eLife* **6**, e26693 (2017).
15. Kim, E. & Hart, T. Improved analysis of CRISPR fitness screens and reduced off-target effects with the BAGEL2 gene essentiality classifier. *Genome Med.* **13**, 2 (2021).
16. Quinlan, A. R. & Hall, I. M. BEDTools: a flexible suite of utilities for comparing genomic features. *Bioinformatics* **26**, 841–842 (2010).
17. Langmead, B. & Salzberg, S. L. Fast gapped-read alignment with Bowtie 2. *Nat. Methods* **9**, 357–359 (2012).
18. Yu, G., Wang, L.-G. & He, Q.-Y. ChIPseeker: an R/Bioconductor package for ChIP peak annotation, comparison and visualization. *Bioinformatics* **31**, 2382–2383 (2015).
19. Martin, M. Cutadapt removes adapter sequences from high-throughput sequencing reads. *EMBnet.journal* **17**, 10–12 (2011).
20. Ramírez, F. *et al.* deepTools2: a next generation web server for deep-sequencing data analysis. *Nucleic Acids Res.* **44**, W160–W165 (2016).
21. Love, M. I., Huber, W. & Anders, S. Moderated estimation of fold change and dispersion for RNA-seq data with DESeq2. *Genome Biol.* **15**, 1–21 (2014).
22. The ENCODE Project Consortium. Expanded encyclopaedias of DNA elements in the human and mouse genomes. *Nature* **583**, 699–710 (2020).
23. Korotkevich, G. *et al.* Fast gene set enrichment analysis. Preprint at https://doi.org/10.1101/060012 (2021).
24. Schindelin, J. *et al.* Fiji: an open-source platform for biological-image analysis. *Nat. Methods* **9**, 676–682 (2012).
25. Kim, D., Paggi, J. M., Park, C., Bennett, C. & Salzberg, S. L. Graph-based genome alignment and genotyping with HISAT2 and HISAT-genotype. *Nat. Biotechnol.* **37**, 907–915 (2019).
26. Robinson, J. T. *et al.* Integrative genomics viewer. *Nat. Biotechnol.* **29**, 24–26 (2011).
27. Ritchie, M. E. *et al.* limma powers differential expression analyses for RNA-sequencing and microarray studies. *Nucleic Acids Res.* **43**, e47 (2015).
28. Zhang, Y. *et al.* Model-based Analysis of ChIP-Seq (MACS). *Genome Biol.* **9**, R137 (2008).
29. Ewels, P., Magnusson, M., Lundin, S. & Käller, M. MultiQC: summarize analysis results for multiple tools and samples in a single report. *Bioinformatics* **32**, 3047–3048 (2016).

30. Patro, R., Duggal, G., Love, M. I., Irizarry, R. A. & Kingsford, C. Salmon provides fast and bias-aware quantification of transcript expression. *Nat. Methods* **14**, 417–419 (2017).

31. Li, H. *et al.* The Sequence Alignment/Map format and SAMtools. *Bioinformatics* **25**, 2078–2079 (2009).

32. Cakir, B. *et al.* Comparison of visualization tools for single-cell RNAseq data. *NAR Genomics Bioinforma.* **2**, lqaa052 (2020).

33. Hao, Y. *et al.* Dictionary learning for integrative, multimodal and scalable single-cell analysis. *Nat. Biotechnol.* **42**, 293–304 (2024).

34. Mölder, F. *et al.* Sustainable data analysis with Snakemake. Preprint at https://doi.org/10.12688/f1000research.29032.1 (2021).

35. Dobin, A. *et al.* STAR: ultrafast universal RNA-seq aligner. *Bioinformatics* **29**, 15–21 (2013).

36. Kassambara, A., Kosinski, M., Biecek, P. survminer: Drawing Survival Curves using ‘ggplot2’. https://rpkgs.datanovia.com/survminer/index.html (2025).

37. Jin, Y., Tam, O. H., Paniagua, E. & Hammell, M. TEtranscripts: a package for including transposable elements in differential expression analysis of RNA-seq datasets. *Bioinformatics* **31**, 3593–3599 (2015).

38. Zerbino, D. R., Johnson, N., Juettemann, T., Wilder, S. P. & Flicek, P. WiggleTools: parallel processing of large collections of genome-wide datasets for visualization and statistical analysis. *Bioinformatics* **30**, 1008–1009 (2014).

39. Perez, G. *et al.* The UCSC Genome Browser database: 2025 update. *Nucleic Acids Res.* **53**, D1243–D1249 (2025).
