# Supplementary material for: VHL synthetic lethality screens uncover CBF-β as a negative regulator of STING
Source: Nat Commun. 2026 Mar 12;17:3841. doi: 10.1038/s41467-026-70517-w (PMC13121600; doi:10.1038/s41467-026-70517-w)
Supplement: Supplementary file 2 — Description of Additional Supplementary Files [file 41467_2026_70517_MOESM2_ESM.pdf]

Description of Additional Supplementary Files

Supplementary Data 1. Analysed CRISPR screen data (BAGEL2, Drug Z, and MAGeCK analysis)

Supplementary Data 2. Analysed RNA sequencing data (gene expression analysis)

Supplementary Data 3. Analysed LC-MS data

Supplementary Data 4. Analysed RNA sequencing data (transposable elements upregulated upon CBF-β knockout)
