# Supplementary material for: VHL synthetic lethality screens uncover CBF-β as a negative regulator of STING
Source: Nat Commun. 2026 Mar 12;17:3841. doi: 10.1038/s41467-026-70517-w (PMC13121600; doi:10.1038/s41467-026-70517-w)
Supplement: Supplementary file 7 — Reporting Summary [file 41467_2026_70517_MOESM7_ESM.pdf]

nature portfolio

Corresponding author(s): James A Nathan

Last updated by author(s): Feb 13, 2026

Reporting Summary

Nature Portfolio wishes to improve the reproducibility of the work that we publish. This form provides structure for consistency and transparency in reporting. For further information on Nature Portfolio policies, see our Editorial Policies and the Editorial Policy Checklist.

Statistics

For all statistical analyses, confirm that the following items are present in the figure legend, table legend, main text, or Methods section.

n/a	Confirmed	
☐	☒	The exact sample size (*n*) for each experimental group/condition, given as a discrete number and unit of measurement
☐	☒	A statement on whether measurements were taken from distinct samples or whether the same sample was measured repeatedly
☐	☒	The statistical test(s) used AND whether they are one- or two-sided<br>*Only common tests should be described solely by name; describe more complex techniques in the Methods section.*
☒	☐	A description of all covariates tested
☒	☐	A description of any assumptions or corrections, such as tests of normality and adjustment for multiple comparisons
☐	☒	A full description of the statistical parameters including central tendency (e.g. means) or other basic estimates (e.g. regression coefficient) AND variation (e.g. standard deviation) or associated estimates of uncertainty (e.g. confidence intervals)
☐	☒	For null hypothesis testing, the test statistic (e.g. *F*, *t*, *r*) with confidence intervals, effect sizes, degrees of freedom and *P* value noted<br>*Give P values as exact values whenever suitable.*
☒	☐	For Bayesian analysis, information on the choice of priors and Markov chain Monte Carlo settings
☒	☐	For hierarchical and complex designs, identification of the appropriate level for tests and full reporting of outcomes
☒	☐	Estimates of effect sizes (e.g. Cohen's *d*, Pearson's *r*), indicating how they were calculated

Our web collection on statistics for biologists contains articles on many of the points above.

Software and code

Policy information about availability of computer code

Data collection	Flow cytometry: FACSDiva v8.0.3<br>qPCR: QuantStudio 7<br>Plate reader: CLARIOstar Plus plate reader<br>Seahorse XF<br>Xenografts: SPECTRAL AMI-HTX<br>Sequencing: HiSeq 4000, NovaSeq 6000, NovaSeq X Plus
Data analysis	Flow cytometry: FlowJo v10<br>CRISPR/Cas9 screening: Cutadapt v4.4, HISAT2 v2.2.1, BAGEL2, MAGeCK, DrugZ, custom pipeline (https://github.com/niekwit/crispr-screens, https://doi.org/10.5281/zenodo.10286661).<br>RNA sequencing: DESeq2, Salmon, STAR v2.7.10b, TEtranscripts v2.2.3, fgsea v1.20.0, custom pipelines (https://github.com/niekwit/rna-seq-salmon-deseq2, https://doi.org/10.5281/zenodo.10139567, https://github.com/niekwit/rna-seq-star-tetranscripts, https://doi.org/10.5281/zenodo.10027278)<br>ChIP sequencing: FastQC v0.12.1, MultiQC v1.21, TrimGalore, Bowtie2 v2.5.3, SAMtools v1.20, BEDTools v2.31.1, SAMtools, Picard v3.1.1, deepTools2, MACS2 v2.2.9.1, WiggleTools v1.2.11, UCSC Genome Browser, GNU Awk v5.0.1, ChIPseeker v1.44.0, IGV v2.16.0, ENCODE, custom pipeline (https://doi.org/10.5281/zenodo.138015265).<br>LC-MS: Peaks 11, limma v3.15<br>TCGA and CPTAC data: survminer v0.4.9<br>Single cell expression data: seurat v5.1.0, sceasy v0.0.7<br>Immunofluorescence: Fiji (ImageJ v1.54p)

R v4.1.2
Python v3.10
Snakemake v8.25.5
GraphPad Prism v9.5.1

For manuscripts utilizing custom algorithms or software that are central to the research but not yet described in published literature, software must be made available to editors and reviewers. We strongly encourage code deposition in a community repository (e.g. GitHub). See the Nature Portfolio guidelines for submitting code & software for further information.

Data

Policy information about availability of data

All manuscripts must include a data availability statement. This statement should provide the following information, where applicable:

- Accession codes, unique identifiers, or web links for publicly available datasets
- A description of any restrictions on data availability
- For clinical datasets or third party data, please ensure that the statement adheres to our policy

Raw data from RNA sequencing, ChIP sequencing and CRISPR/Cas9 screens have been deposited at GEO and are publicly available at GSE300828 (https://www.ncbi.nlm.nih.gov/geo/query/acc.cgi?acc=GSE300828), GSE270775 (https://www.ncbi.nlm.nih.gov/geo/query/acc.cgi?acc=GSE270775), and GSE270776 (https://www.ncbi.nlm.nih.gov/geo/query/acc.cgi?acc=GSE270776). Raw mass spectrometry data have been deposited at ProteomeXchange and are publicly available at PXD074426 (https://www.ebi.ac.uk/pride/archive/projects/PXD074426). Source Data are provided with this paper.

Research involving human participants, their data, or biological material

Policy information about studies with human participants or human data. See also policy information about sex, gender (identity/presentation), and sexual orientation and race, ethnicity and racism.

Reporting on sex and gender	This study does not report any new data involving human participants.
Reporting on race, ethnicity, or other socially relevant groupings	This study does not report any new data involving human participants.
Population characteristics	This study does not report any new data involving human participants.
Recruitment	This study does not report any new data involving human participants.
Ethics oversight	This study does not report any new data involving human participants.

Note that full information on the approval of the study protocol must also be provided in the manuscript.

Field-specific reporting

Please select the one below that is the best fit for your research. If you are not sure, read the appropriate sections before making your selection.

[x] Life sciences [ ] Behavioural & social sciences [ ] Ecological, evolutionary & environmental sciences

For a reference copy of the document with all sections, see nature.com/documents/nr-reporting-summary-flat.pdf

Life sciences study design

All studies must disclose on these points even when the disclosure is negative.

Sample size	No sample size calculations were performed, but sample sizes were determined by existing studies in the field to enable reproducibility. All stages of CRISPR/Cas9 screening were performed to maintain at least 400-fold (786O screen) or 500-fold (RCC4 screen) coverage of the library. Experiments are representative of a minimum of three (or in rare cases two) biologically independent replicates.
Data exclusions	No data were excluded from the study.
Replication	Major hits from CRISPR/Cas9 screens were replicated in individual CRISPR/Cas9 and shRNA experiments. Data shown for other experiments are generally representative of multiple independent repeats, with specific details given in the methods and figure legends. All attempts at replication were successful.
Randomization	Samples were generally allocated into experimental groups in the order displayed in the manuscript as systemic bias was not felt to affect these experiments. For xenograft experiments, mice were randomly assigned into groups. Flow cytometry and immunofluorescence analyses were performed in a random order.
Blinding	Investigators were blinded during both data collection and analysis for experiments susceptible to bias, including murine xenografts, competitive growth assays, γ-H2A.X foci determination by immunofluorescence, and the quantification of clonogenic assays. Samples for RNA-Seq and ChIP-Seq were processed in a blinded format. Blinding was not feasible for other assays, such as immunoblots, as these required sample presentation in the given order to permit effective data presentation.

Behavioural & social sciences study design

All studies must disclose on these points even when the disclosure is negative.

Study description	*Briefly describe the study type including whether data are quantitative, qualitative, or mixed-methods (e.g. qualitative cross-sectional, quantitative experimental, mixed-methods case study).*
Research sample	*State the research sample (e.g. Harvard university undergraduates, villagers in rural India) and provide relevant demographic information (e.g. age, sex) and indicate whether the sample is representative. Provide a rationale for the study sample chosen. For studies involving existing datasets, please describe the dataset and source.*
Sampling strategy	*Describe the sampling procedure (e.g. random, snowball, stratified, convenience). Describe the statistical methods that were used to predetermine sample size OR if no sample-size calculation was performed, describe how sample sizes were chosen and provide a rationale for why these sample sizes are sufficient. For qualitative data, please indicate whether data saturation was considered, and what criteria were used to decide that no further sampling was needed.*
Data collection	*Provide details about the data collection procedure, including the instruments or devices used to record the data (e.g. pen and paper, computer, eye tracker, video or audio equipment) whether anyone was present besides the participant(s) and the researcher, and whether the researcher was blind to experimental condition and/or the study hypothesis during data collection.*
Timing	*Indicate the start and stop dates of data collection. If there is a gap between collection periods, state the dates for each sample cohort.*
Data exclusions	*If no data were excluded from the analyses, state so OR if data were excluded, provide the exact number of exclusions and the rationale behind them, indicating whether exclusion criteria were pre-established.*
Non-participation	*State how many participants dropped out/declined participation and the reason(s) given OR provide response rate OR state that no participants dropped out/declined participation.*
Randomization	*If participants were not allocated into experimental groups, state so OR describe how participants were allocated to groups, and if allocation was not random, describe how covariates were controlled.*

Ecological, evolutionary & environmental sciences study design

All studies must disclose on these points even when the disclosure is negative.

Study description	*Briefly describe the study. For quantitative data include treatment factors and interactions, design structure (e.g. factorial, nested, hierarchical), nature and number of experimental units and replicates.*
Research sample	*Describe the research sample (e.g. a group of tagged Passer domesticus, all Stenocereus thurberi within Organ Pipe Cactus National Monument), and provide a rationale for the sample choice. When relevant, describe the organism taxa, source, sex, age range and any manipulations. State what population the sample is meant to represent when applicable. For studies involving existing datasets, describe the data and its source.*
Sampling strategy	*Note the sampling procedure. Describe the statistical methods that were used to predetermine sample size OR if no sample-size calculation was performed, describe how sample sizes were chosen and provide a rationale for why these sample sizes are sufficient.*
Data collection	*Describe the data collection procedure, including who recorded the data and how.*
Timing and spatial scale	*Indicate the start and stop dates of data collection, noting the frequency and periodicity of sampling and providing a rationale for these choices. If there is a gap between collection periods, state the dates for each sample cohort. Specify the spatial scale from which the data are taken*
Data exclusions	*If no data were excluded from the analyses, state so OR if data were excluded, describe the exclusions and the rationale behind them, indicating whether exclusion criteria were pre-established.*
Reproducibility	*Describe the measures taken to verify the reproducibility of experimental findings. For each experiment, note whether any attempts to repeat the experiment failed OR state that all attempts to repeat the experiment were successful.*
Randomization	*Describe how samples/organisms/participants were allocated into groups. If allocation was not random, describe how covariates were controlled. If this is not relevant to your study, explain why.*
Blinding	*Describe the extent of blinding used during data acquisition and analysis. If blinding was not possible, describe why OR explain why blinding was not relevant to your study.*

Did the study involve field work? ☐ Yes ☐ No

Field work, collection and transport

Field conditions	*Describe the study conditions for field work, providing relevant parameters (e.g. temperature, rainfall).*
Location	*State the location of the sampling or experiment, providing relevant parameters (e.g. latitude and longitude, elevation, water depth).*
Access & import/export	*Describe the efforts you have made to access habitats and to collect and import/export your samples in a responsible manner and in compliance with local, national and international laws, noting any permits that were obtained (give the name of the issuing authority, the date of issue, and any identifying information).*
Disturbance	*Describe any disturbance caused by the study and how it was minimized.*

Reporting for specific materials, systems and methods

We require information from authors about some types of materials, experimental systems and methods used in many studies. Here, indicate whether each material, system or method listed is relevant to your study. If you are not sure if a list item applies to your research, read the appropriate section before selecting a response.

Materials & experimental systems

n/a	Involved in the study	
☐	☒	Antibodies
☐	☒	Eukaryotic cell lines
☒	☐	Palaeontology and archaeology
☐	☒	Animals and other organisms
☒	☐	Clinical data
☒	☐	Dual use research of concern
☒	☐	Plants

Methods

n/a	Involved in the study	
☐	☒	ChIP-seq
☐	☒	Flow cytometry
☒	☐	MRI-based neuroimaging

Antibodies

Antibodies used

Details of primary and secondary antibodies are described in Supplementary Table 1 and below:
Rat anti-BrdU - Abcam - Cat#ab6326
Rabbit anti-CBF-β - Cell Signaling Technology - Cat#62184
Rabbit anti-CBF-β - Diagenode - Cat#C15310002
Rabbit anti-cGAS - Cell Signaling Technology - Cat#D1D3G
Mouse anti-FLAG M2 - Sigma - Cat#F1804
Mouse anti-GFP - Roche - Cat#11814460001
Rabbit anti-H3K36me3 - Cell Signaling Technology - Cat#9763
Mouse anti-HIF-1α - BD Biosciences - Cat#610959
Mouse anti-HIF-1β - Cell Signaling Technology - Cat#5537S
Rabbit anti-IFI44 - ThermoFisher - Cat#PA5-65370
Mouse anti-IRF3 - BioLegend - Cat#655704
Rabbit anti-IRF3 - Cell Signaling Technology - Cat#11904
Rabbit anti-IRF7 - Cell Signaling Technology - Cat#13014
Rabbit anti-IRF9 - Cell Signaling Technology - Cat#76684
Rabbit anti-ISG15 - Santa Cruz - Cat#50366
Rabbit anti-KEAP1 - Cell Signaling Technology - Cat#4678
Mouse anti-MDA5 - Hertzog et al.
Rabbit anti-MX1 - Cell Signaling Technology - Cat#37849
Rabbit anti-NRF2 - Cell Signaling Technology - Cat#12721
Rabbit anti-PARP1 - Cell Signaling Technology - Cat#9542S
Rabbit anti-Phospho-Histone 2A.X (Ser139) - Cell Signaling Technology - Cat#9718
Rabbit anti-Phospho-IRF3 (Ser386) - Cell Signaling Technology - Cat#37829
Rabbit anti-Phospho-STAT1 (Tyr701) - Cell Signaling Technology - Cat#9167
Rabbit anti-RUNX1 - Cell Signaling Technology - Cat#4344
Rabbit anti-RUNX2 - Cell Signaling Technology - Cat#8486
Rabbit anti-STING - Cell Signaling Technology - Cat#13647
Goat anti-Rabbit Alexa-Fluor 488 IgG - Invitrogen - Cat#A11034
Goat anti-Rabbit Alexa-Fluor 647 IgG- Invitrogen - Cat#A21245
Goat anti-Rat Alexa-Fluor 647 IgG - Invitrogen - Cat#A21247
Goat anti-Mouse Peroxidase-AffiniPure IgG - Jackson - Cat#115-035-146
Goat anti-Rabbit Peroxidase-AffiniPure IgG - Jackson - Cat#111-035-045

Validation

Validation data for all the antibodies can be found at the manufacturer's websites:
- https://www.abcam.com/en-us/products/primary-antibodies/brdu-antibody-bu1-75-icr1-proliferation-marker-ab6326?srsltid=AfmBOor0tE3oA6RGXTzw1Sc4ae7jIEd3U3h386jqWHJNwH-K75yZBosT
- https://www.cellsignal.com/products/primary-antibodies/cbfb-d4n2n-rabbit-mab/62184?srsltid=AfmBOoqjX4lnybcKdFsYUcgjOQMTbBXlc4MrKXpslHazxEaZHVBeolg2
- https://www.diagenode.com/en/p/cbfb-polyclonal-antibody-classic-100-ul
- https://www.cellsignal.com/products/primary-antibodies/cgas-d1d3g-rabbit-mab/15102?srsltid=AfmBOoozvGosiB5-Vl9_cA2k1O_XCwsGCOL_5vPJWomQAQc8Nv-OvVv8
- https://www.sigmaaldrich.com/GB/en/product/sigma/f1804?srsltid=AfmBOooFEQcZ19BNPjkue2zqGBaqJtCqLf8uWFR9xueAax_mRF6LCGOC
- https://www.sigmaaldrich.com/GB/en/product/roche/11814460001?srsltid=AfmBOop1_DNYqmv9H9q9K2UUkI0DUQVvMdSkF0XfDvm4r08sIyFk_ZYc
- https://www.cellsignal.com/products/primary-antibodies/tri-methyl-histone-h3-lys36-antibody/9763?srsltid=AfmBOooIDoZzGBbSdgFGSDfjJPbcC2EeRIa875e3GUqGz3XmfeXWKzjV
- https://www.bdbiosciences.com/en-gb/products/reagents/microscopy-imaging-reagents/immunofluorescence-reagents/purified-mouse-anti-human-hif-1.610959?tab=product_details
- https://www.cellsignal.com/products/primary-antibodies/hif-1b-arnt-d28f3-xp-rabbit-mab/5537?srsltid=AfmBOoozguLWQ2oy6FhmSWPJ8QhQgwh2fTFPkUEOKKwrW1ZpsHg4PFtA
- https://www.thermofisher.com/antibody/product/IFI44-Antibody-Polyclonal/PA5-65370
- https://www.biolegend.com/en-gb/products/purified-anti-irf3-antibody-8629
- https://www.cellsignal.com/products/primary-antibodies/irf-3-d6i4c-xp-rabbit-mab/11904?srsltid=AfmBOoppdhVks7p0fpYHkqhBeIsxtd5pSXdwYuRHKZIzQ1dnYB7vMrsu
- https://www.cellsignal.com/products/primary-antibodies/irf-7-d2a1j-rabbit-mab/13014?srsltid=AfmBOoq1-uvyIs5L9EQ6VxkFnIeogR9JF-E0dFqoiTRgCf5i5ZyakMnE
- https://www.cellsignal.com/products/primary-antibodies/irf-9-d2t8m-rabbit-mab/76684?srsltid=AfmBOorZ8BmWIk11PpTabuASSuMNNkIpqyLGwlJrm92TfWR5X-Gb80fa
- https://www.scbt.com/p/isg15-antibody-h-150?srsltid=AfmBOoqGrSsNcQf25M-CguBlFiJDNmXAd7wGak6rZ1cupGLxL1B_CsZj
- https://www.cellsignal.com/products/primary-antibodies/keap1-p586-antibody/4678
- https://www.cellsignal.com/products/primary-antibodies/mx1-d3w7i-rabbit-mab/37849?srsltid=AfmBOoopXxFH1UtAte-3rdl7l0f-l73zxhMghvv6RUI-O-CLdhWQ-Izt
- https://www.cellsignal.com/products/primary-antibodies/nrf2-d1z9c-xp-rabbit-mab/12721
- https://www.cellsignal.com/products/primary-antibodies/parp-antibody/9542?srsltid=AfmBOoqIt_nErIK9n55vExu8uB46CJEXo9A5WnIpn6h5utc_GIUL-FbE
- https://www.cellsignal.com/products/primary-antibodies/phospho-histone-h2a-x-ser139-20e3-rabbit-mab/9718?srsltid=AfmBOoqwfFzF5Pzvg13pj074MsqJphtI6jIkN0HP3wPBIxa5uArQCWBB
- https://www.cellsignal.com/products/primary-antibodies/phospho-irf-3-ser386-e7j8g-xp-rabbit-mab/37829?srsltid=AfmBOop-onTm5j6UY9BGIzO_ioo5NWBgAYK3U6MmEX8oAR4wuslEM-c1
- https://www.cellsignal.com/products/primary-antibodies/phospho-stat1-tyr701-58d6-rabbit-mab/9167?srsltid=AfmBOopsaOxLUaZAQBYK3FzAQv3eIQyYiUOfZ0hfOPl0WX0zzbv98xDa
- https://www.cellsignal.com/products/primary-antibodies/aml1-antibody/4334?srsltid=AfmBOopiqInfT-9vXSmXf6fH9ojhqiW6SaJ3F59uNWxcvpu0g51LH1Ue
- https://www.cellsignal.com/products/primary-antibodies/runx2-d1h7-rabbit-mab/8486?srsltid=AfmBOooq7Ikb9XlodYrVVtoH48eJVkpS__HICmcD5FzjUBbWegqhrWEb
- https://www.cellsignal.com/products/primary-antibodies/sting-d2p2f-rabbit-mab/13647?srsltid=AfmBOoq97Rjw98G1gTqhrom81NzCvHmzk8oSpPKUnuXa_8OtklxXAwrK
- https://www.thermofisher.com/antibody/product/Goat-anti-Rabbit-IgG-H-L-Highly-Cross-Adsorbed-Secondary-Antibody-Polyclonal/A-11034
- https://www.thermofisher.com/antibody/product/Goat-anti-Rabbit-IgG-H-L-Highly-Cross-Adsorbed-Secondary-Antibody-Polyclonal/A-21245
- https://www.thermofisher.com/antibody/product/Goat-anti-Rat-IgG-H-L-Cross-Adsorbed-Secondary-Antibody-Polyclonal/A-21247
- https://www.jacksonimmuno.com/catalog/products/115-035-146
- https://www.jacksonimmuno.com/catalog/products/111-035-045

Eukaryotic cell lines

Policy information about cell lines and Sex and Gender in Research

Cell line source(s)

Cell lines were sourced as detailed in Supplementary Table 1 and below:
A498 - P. Schraml lab
HEK293T - ATCC - Cat#CRL-3216
HKC8 - P.H. Maxwell lab
HK2 - ATCC - Cat#CRL-2190
RCC4 - P.H. Maxwell lab
RCC4+VHL - P.J. Ratcliffe lab
RCC10 - P.H. Maxwell lab
786O - ATCC - Cat#CRL-1932
786O+VHL - W.G. Kaelin Jr. lab
769P - P. Schraml lab

Authentication

All cell lines were authenticated by short tandem repeat profiling (Eurofins Genomics).

Mycoplasma contamination	All cell lines tested negative for Mycoplasma contamination.
Commonly misidentified lines (See ICLAC register)	No commonly misidentified cell lines were used.

Palaeontology and Archaeology

Specimen provenance	*Provide provenance information for specimens and describe permits that were obtained for the work (including the name of the issuing authority, the date of issue, and any identifying information). Permits should encompass collection and, where applicable, export.*
Specimen deposition	*Indicate where the specimens have been deposited to permit free access by other researchers.*
Dating methods	*If new dates are provided, describe how they were obtained (e.g. collection, storage, sample pretreatment and measurement), where they were obtained (i.e. lab name), the calibration program and the protocol for quality assurance OR state that no new dates are provided.*

☐ Tick this box to confirm that the raw and calibrated dates are available in the paper or in Supplementary Information.

Ethics oversight	*Identify the organization(s) that approved or provided guidance on the study protocol, OR state that no ethical approval or guidance was required and explain why not.*

Note that full information on the approval of the study protocol must also be provided in the manuscript.

Animals and other research organisms

Policy information about studies involving animals; ARRIVE guidelines recommended for reporting animal research, and Sex and Gender in Research

Laboratory animals	Subcutaneous xenograft: 8 week old female NSG mice (NOD.Cg-Prkdcscid Il2rgtm1Wjl/SzJ (Charles River RRID:IMSR_JAX:005557)). Orthotopic xenograft: 6-8 week old male and female NSG mice (UTSW Animal Resource Center).
Wild animals	The study did not involve wild animals.
Reporting on sex	Data were not disaggregated for sex as experiments were not powered to provide discrimination of sex-based differences in tumour growth.
Field-collected samples	The study did not involve samples collected from the field.
Ethics oversight	Animal experiments were performed according to protocols approved by either the University of Cambridge Animal Welfare and Ethical Review Board in compliance with the Animals (Scientific Procedures) Act 1986 and UK Home Office regulations, or by the Institutional Animal Care and Use Committee of UT Southwestern Medical Center following NIH guidelines.

Note that full information on the approval of the study protocol must also be provided in the manuscript.

Clinical data

Policy information about clinical studies

All manuscripts should comply with the ICMJE guidelines for publication of clinical research and a completed CONSORT checklist must be included with all submissions.

Clinical trial registration	*Provide the trial registration number from ClinicalTrials.gov or an equivalent agency.*
Study protocol	*Note where the full trial protocol can be accessed OR if not available, explain why.*
Data collection	*Describe the settings and locales of data collection, noting the time periods of recruitment and data collection.*
Outcomes	*Describe how you pre-defined primary and secondary outcome measures and how you assessed these measures.*

Dual use research of concern

Policy information about dual use research of concern

Hazards

Could the accidental, deliberate or reckless misuse of agents or technologies generated in the work, or the application of information presented in the manuscript, pose a threat to:

No	Yes	
☐	☐	Public health
☐	☐	National security
☐	☐	Crops and/or livestock
☐	☐	Ecosystems
☐	☐	Any other significant area

Experiments of concern

Does the work involve any of these experiments of concern:

No	Yes	
☐	☐	Demonstrate how to render a vaccine ineffective
☐	☐	Confer resistance to therapeutically useful antibiotics or antiviral agents
☐	☐	Enhance the virulence of a pathogen or render a nonpathogen virulent
☐	☐	Increase transmissibility of a pathogen
☐	☐	Alter the host range of a pathogen
☐	☐	Enable evasion of diagnostic/detection modalities
☐	☐	Enable the weaponization of a biological agent or toxin
☐	☐	Any other potentially harmful combination of experiments and agents

Plants

Seed stocks: N/A

Novel plant genotypes: N/A

Authentication: N/A

ChIP-seq

Data deposition

☒ Confirm that both raw and final processed data have been deposited in a public database such as GEO.

☒ Confirm that you have deposited or provided access to graph files (e.g. BED files) for the called peaks.

Data access links
May remain private before publication.

https://www.ncbi.nlm.nih.gov/geo/query/acc.cgi?acc=GSE300828
Token: kpoxqwiejbenjqn

Files in database submission

EV_1_in_R1_001.fastq.gz
EV_1_in_R2_001.fastq.gz
EV_1_R1_001.fastq.gz
EV_1_R2_001.fastq.gz
EV_2_in_R1_001.fastq.gz
EV_2_in_R2_001.fastq.gz
EV_2_R1_001.fastq.gz
EV_2_R2_001.fastq.gz
EV_3_in_R1_001.fastq.gz
EV_3_in_R2_001.fastq.gz
EV_3_R1_001.fastq.gz
EV_3_R2_001.fastq.gz
OE_1_in_R1_001.fastq.gz
OE_1_in_R2_001.fastq.gz
OE_1_R1_001.fastq.gz
OE_1_R2_001.fastq.gz
OE_3_in_R1_001.fastq.gz
OE_3_in_R2_001.fastq.gz

OE_3_R1_001.fastq.gz
OE_3_R2_001.fastq.gz
EV_1.bw
EV_1_in.bw
EV_2.bw
EV_2_in.bw
EV_3.bw
EV_3_in.bw
OE_1.bw
OE_1_in.bw
OE_3.bw
OE_3_in.bw
EV.bed
OE.bed

Genome browser session (e.g. UCSC)

N/A

Methodology

Replicates

n=3 biologically independent replicates (786O Cas9 cells transduced with empty vector), n=2 biologically independent replicates (786O Cas9 cells transduced with CBF-β-FLAG vector). Replicates showed high concordance in PCA analysis.

Sequencing depth

50bp paired-end reads. Each post-alignment, deduplicated sample consisted of at least 100M fragments.

Antibodies

Rabbit anti-CBF-β (Diagenode Cat#C15310002)

Peak calling parameters

MACS2 --broad --broad-cutoff 0.05

Data quality

Data quality was first determined with FastQC/MultiQC. The EV consensus peak set consists of 12 peak w. FDR < 0.05 with one peak > 5-fold enrichment over input, while the OE sample consensus peak set consists of 47 peaks with FDR < 0.05, with 5 peaks displaying 5-fold enrichment over the input.

Software

A Snakemake v8.25.5 pipeline was used to analyse ChIP-Seq data (https://doi.org/10.5281/zenodo.138015265): read quality was first assessed with FastQC v0.12.1 and MultiQC v1.21, after which the reads were quality trimmed with TrimGalore. Alignment against the human genome (hg38, build 113) was performed using Bowtie2 v2.5.3. Using SAMtools view v1.20, only fragments with MAPQ > 10 were retained for further analysis. BEDTools intersect v2.31.1 was then used to remove blacklisted regions (obtained from ENCODE, https://www.encodeproject.org/files/ENCFF356LFX). To remove duplicates, fragments were first sorted by coordinate using SAMtools sort, after which Picard MarkDuplicated v3.1.1 was used with the command line flag --REMOVE_DUPLICATES true. The resulting BAM file was indexed using the SAMtools index command. BigWig files for individual replicates were made with deepTools bamCoverage v3.5.5 with the settings binSize 10 and normalizeUsing RPKM. The Principal Component Analysis of these BigWig files was performed with deepTools multiBigWigSummary and deepTools plotPCA, while plotting was done with a custom R script. Peak calling on individual replicates was performed on the deduplicated BAM files with MACS2 callpeak v2.2.9.1 (broad peak calling) with q=0.05 and broad cutoff=0.05 and the matched input sample as a control. Consensus peaks (BED format) were generated using a custom Python script that identified regions of overlap between all replicates.

To create the coverage plot, mean BigWig files were first generated from all replicates of each condition (IP and input): first a mean Wig file was created using WiggleTools v1.2.11, which was converted to the BigWig format using wigToBigWig v4 (UCSC Genome Browser). To compute the ratio of IP over input, deeptools bigwigCompare was used with the command line flags --operation ratio --skipZeroOverZero. bigWigAverageOverBed v469 (UCSC Genome Browser) was used with the consensus peak file described above to obtain the average ratio of IP over input in the consensus peaks. GNU Awk v5.0.1 was then used to append these scores as an additional column to the consensus peak BED file. Finally, this scored BED file was used as input for the coverage plot using a custom R script that utilised the Bioconductor package ChIPseeker v1.44.0. Genome tracks were visualised with IGV v2.16.0.

Flow Cytometry

Plots

Confirm that:

- [x] The axis labels state the marker and fluorochrome used (e.g. CD4-FITC).
- [x] The axis scales are clearly visible. Include numbers along axes only for bottom left plot of group (a 'group' is an analysis of identical markers).
- [x] All plots are contour plots with outliers or pseudocolor plots.
- [x] A numerical value for number of cells or percentage (with statistics) is provided.

Methodology

Sample preparation

Following appropriate fixation and staining, as detailed in the methods, cells were collected in 5 ml FACS tubes, centrifuged, washed and resuspended in PBS.

Instrument

Fortessa flow cytometer (BD Biosciences)

Software	Collection: FACSDiva v.8.0.3 (BD Biosciences)<br>Analysis: FlowJo v10 (BD Biosciences)
Cell population abundance	No cell sorting was performed.
Gating strategy	Cells were gated using forward and side scatter parameters, then for doublet discrimination using forward scatter-width and -area. Gating strategies for individual experiments are provided in Supplementary Fig. 2.

☒ Tick this box to confirm that a figure exemplifying the gating strategy is provided in the Supplementary Information.

Magnetic resonance imaging

Experimental design

Design type	*Indicate task or resting state; event-related or block design.*
Design specifications	*Specify the number of blocks, trials or experimental units per session and/or subject, and specify the length of each trial or block (if trials are blocked) and interval between trials.*
Behavioral performance measures	*State number and/or type of variables recorded (e.g. correct button press, response time) and what statistics were used to establish that the subjects were performing the task as expected (e.g. mean, range, and/or standard deviation across subjects).*

Acquisition

Imaging type(s)	*Specify: functional, structural, diffusion, perfusion.*
Field strength	*Specify in Tesla*
Sequence & imaging parameters	*Specify the pulse sequence type (gradient echo, spin echo, etc.), imaging type (EPI, spiral, etc.), field of view, matrix size, slice thickness, orientation and TE/TR/flip angle.*
Area of acquisition	*State whether a whole brain scan was used OR define the area of acquisition, describing how the region was determined.*

Diffusion MRI ☐ Used ☐ Not used

Preprocessing

Preprocessing software	*Provide detail on software version and revision number and on specific parameters (model/functions, brain extraction, segmentation, smoothing kernel size, etc.).*
Normalization	*If data were normalized/standardized, describe the approach(es): specify linear or non-linear and define image types used for transformation OR indicate that data were not normalized and explain rationale for lack of normalization.*
Normalization template	*Describe the template used for normalization/transformation, specifying subject space or group standardized space (e.g. original Talairach, MNI305, ICBM152) OR indicate that the data were not normalized.*
Noise and artifact removal	*Describe your procedure(s) for artifact and structured noise removal, specifying motion parameters, tissue signals and physiological signals (heart rate, respiration).*
Volume censoring	*Define your software and/or method and criteria for volume censoring, and state the extent of such censoring.*

Statistical modeling & inference

Model type and settings	*Specify type (mass univariate, multivariate, RSA, predictive, etc.) and describe essential details of the model at the first and second levels (e.g. fixed, random or mixed effects; drift or auto-correlation).*
Effect(s) tested	*Define precise effect in terms of the task or stimulus conditions instead of psychological concepts and indicate whether ANOVA or factorial designs were used.*

Specify type of analysis: ☐ Whole brain ☐ ROI-based ☐ Both

Statistic type for inference<br>(See Eklund et al. 2016)	*Specify voxel-wise or cluster-wise and report all relevant parameters for cluster-wise methods.*
Correction	*Describe the type of correction and how it is obtained for multiple comparisons (e.g. FWE, FDR, permutation or Monte Carlo).*

Models & analysis

n/a	Involved in the study
☐	☐ Functional and/or effective connectivity
☐	☐ Graph analysis
☐	☐ Multivariate modeling or predictive analysis

Functional and/or effective connectivity	*Report the measures of dependence used and the model details (e.g. Pearson correlation, partial correlation, mutual information).*
Graph analysis	*Report the dependent variable and connectivity measure, specifying weighted graph or binarized graph, subject- or group-level, and the global and/or node summaries used (e.g. clustering coefficient, efficiency, etc.).*
Multivariate modeling and predictive analysis	*Specify independent variables, features extraction and dimension reduction, model, training and evaluation metrics.*
